# Unveiling the Molecular Crosstalk Between Periodontal and Cardiovascular Diseases: A Systematic Review

**DOI:** 10.3390/dj13030098

**Published:** 2025-02-25

**Authors:** Gunaraj Dhungana, Dollada Srisai, Chethan Sampath, Jeremiah Soliman, Regan M. Kelly, Honar Y. Saleh, Abdelrahman Sedik, Edilberto Raynes, Alexys Ferguson, Leela Subhashini Choudary Alluri, Pandu R. Gangula

**Affiliations:** 1 Department of Oral Diagnostic Sciences & Research, School of Dentistry, Meharry Medical College, Nashville, TN 37208, USA; gunaraj.dhungana1@mmc.edu (G.D.); dsrisai@mmc.edu (D.S.); csampath@mmc.edu (C.S.); jsoliman23@mmc.edu (J.S.); hsaleh23@mmc.edu (H.Y.S.); asedik23@mmc.edu (A.S.); afkelly@mmc.edu (A.F.); 2 Department of Professional and Medical Education, School of Medicine, Meharry Medical College, Nashville, TN 37208, USA; eraynes@mmc.edu; 3 Department of Periodontology, School of Dentistry, Meharry Medical College, Nashville, TN 37208, USA; lalluri@mmc.edu

**Keywords:** periodontal disease, oral microbiota, red complex bacteria, cardiovascular disease, endothelial dysfunction, systemic inflammation

## Abstract

**Background/Objectives:** Periodontal disease (PD) is a chronic inflammatory condition caused by dysbiosis of the oral microbiome. PD is linked to systemic inflammation and endothelial dysfunction, which associate it with cardiovascular disease (CVD). This systematic review explores the molecular and microbial mechanisms through which periodontal pathogens, including “Red Complex” bacteria (*Porphyromonas gingivalis*, *Tannerella forsythia*, *Treponema denticola*) and *Fusobacterium nucleatum*, influence cardiovascular health via inflammatory pathways, immune modulation, and microbial dissemination. **Methods**: A systematic review was conducted following PRISMA guidelines. A literature search was conducted in the PubMed and ScienceDirect databases using relevant keywords, with strict inclusion and exclusion criteria, from the first week of September 2024 to the first week of October 2024. Studies addressing the relationship between PD and CVD were assessed for methodological rigor, relevance, and data availability. The outcomes were synthesized using a descriptive narrative approach. Out of 591 records screened, 421 full-text articles were sought for retrieval. The final review included 58 articles providing supplementary aggregated data after eligibility assessment. **Results:** The pathogenesis of PD involves the activation of immune cells and the release of pro-inflammatory cytokines (such as IL-1, IL-6, TNF-α, and PGE2) and chemokines (including IL-8 and MCP-1) along with oxidative stress driven by reactive oxygen species (ROS). Periodontal pathogens trigger endothelial oxidative stress and systemic inflammation via Toll-like receptors (TLRs), NF-κB signaling, and nitric oxide (NO) dysregulation, contributing to endothelial dysfunction and atherogenesis. Biomarkers, such as C-reactive protein, interleukins, and matrix metalloproteinases (MMPs), further highlight the systemic inflammatory response. **Conclusions:** This review underscores the significant role of periodontal pathogens and inflammatory mediators in systemic health, particularly in the progression of CVD. Although existing evidence illustrates these associations, the underlying molecular mechanisms remain inadequately understood, indicating a need for further research to advance precision medicine and therapeutic strategies.

## 1. Introduction

The equilibrium of the oral microbiota is essential for maintaining host health, homeostasis, and immunity. There are approximately 700 species in this complex microbial community, classified into 185 genera and 12 phyla, including *Firmicutes*, *Fusobacteria*, *Actinobacteria*, *Proteobacteria*, *Bacteroidetes*, *Chlamydiae*, *Chloroflexi*, and *Spirochaetes*. The interactions between these microorganisms and their hosts are critical for maintaining oral health in commensal, symbiotic, and pathogenic forms [1]. A reduction in beneficial bacteria with an increase in pathogenic ones can disrupt the oral microbial balance, leading to oral dysbiosis. This dysbiosis can result from factors such as poor oral hygiene, diet, antibiotic use, and underlying health conditions. Conversely, dysbiosis can lead to an overgrowth of opportunistic pathogens, such as *Porphyromonas gingivalis* (*P. gingivalis*), *Tannerella forsythia* (*T. forsythia*), *Treponemma denticola* (*T. denticola*), *Aggregatibacter actinomycetemcomitans* (*A. actinomycetemcomitans*), *Prevotella intermedia* (*P. intermedia*), *Fusobacterium nucleatum* (*F. nucleatum*), *Eikenella corredens* (*E. corredens*), *Candida albicans* (*C. albicans*), etc., which are implicated in the development of oral diseases such as dental caries, periodontal disease (PD), oral candidiasis, etc. [2]. The “Red Complex” bacteria, including *P. gingivalis*, *T. forsythia*, and *T. denticola*, are particularly implicated in PD due to their virulence, ability to subvert immune responses, and synergistic interactions within the biofilm [3]. Furthermore, oral microbial dysbiosis is not only confined to local infections but can also contribute to systemic health complications. Oral pathogens can spread from the subgingival biofilm into the bloodstream, allowing for them to reach various organs and tissues [4]. This dissemination may play a role in the development of multiple systemic diseases by contributing to inflammation and pathogenic processes beyond the oral cavity [5]. These include respiratory diseases such as asthma, gastrointestinal disorders like inflammatory bowel disease, cardiovascular diseases, diabetes, obesity, metabolic disorders, autoimmune diseases, Alzheimer’s disease, and certain cancers [6]. The mechanisms underlying these associations involve complex interactions between oral pathogens, host immune responses, and systemic inflammation.

This review highlights the global prevalence of periodontal disease (PD) and its significant role in contributing to systemic inflammation, with a particular focus on endothelial dysfunction and its implications in the pathogenesis of cardiovascular disease (CVD). The primary objective of this systematic review is to elucidate the association between PD and CVD, delving into the molecular and microbial mechanisms through which periodontal pathogens, notably the “Red Complex” bacteria—*P. gingivalis*, *T. forsythia*, and *T. denticola*—as well as *F. nucleatum*, exert their influence on cardiovascular health.

## 2. Materials and Methods

This systematic review was conducted in accordance with the PRISMA guidelines to investigate the relationship between periodontal disease, the oral microbiome, and cardiovascular disease, with a specific focus on the underlying mechanisms by which pathogenic oral bacteria contribute to cardiovascular conditions [7]. This study was submitted and accepted in the INPLASY database (Reg. No. INPLASY202510055). The PICO method was employed to answer the research question as stated below:

P (Population/Problem): Individuals with periodontal disease (PD), with or without cardiovascular disease (CVD).

I (Intervention/Exposure): Oral microbiome dysbiosis and the presence of periodontal pathogens contributing to systemic inflammation, immune modulation, endothelial dysfunction, and microbial dissemination.

C (Comparison): Individuals with or without periodontal disease and cardiovascular disease.

O (Outcome): Systemic inflammation and endothelial dysfunction as mechanisms linking PD to CVD. Molecular and microbial mechanisms, including inflammatory pathways, immune modulation, and microbial dissemination, affecting vascular function.

The literature search strategy utilized the Medical Subject Headings (MeSH) term and additionally employed a combination of keywords, such as periodontitis, oral microbiome, cardiovascular diseases, underlying mechanisms, atherosclerosis, and red complex bacteria. The literature search was performed in two major databases: PubMed and ScienceDirect. The screening process began with an evaluation of titles and abstracts to determine the relevance of the articles to the research objectives. The initial screening was conducted by two reviewers from the first week of September 2024 to the first week of October 2024. The details of the electronic search strategy are provided in the Supplementary Material.

Inclusion Criteria: To ensure the inclusion of relevant and high-quality studies, the following eligibility criteria were applied:

1. Studies must explicitly examine the association between periodontal disease and cardiovascular disease, focusing on mechanisms linking pathogenic oral bacteria to the development of cardiovascular conditions.
2. Sufficient data must be available for extraction and analysis.
3. Articles must be peer-reviewed, written in English, and published within the last decade.
4. Original studies must be designed as observational, cross-sectional, prospective longitudinal, retrospective studies, and in vitro/in vivo studies.

Exclusion Criteria:

1. Irrelevant topics, such as studies not focused on periodontal disease, cardiovascular disease, or their relationship.
2. Non-rodent models: Studies conducted in species other than rodents.
3. Irrelevant outcomes: Studies that did not report relevant outcomes related to cardiovascular health.
4. Unrelated systemic conditions: Studies addressing systemic conditions not directly linked to periodontal disease or cardiovascular disease.

Titles and abstracts of all retrieved articles were independently screened by two reviewers to assess their relevance to the research objectives. Full-text articles were obtained for studies meeting the inclusion criteria, and eligibility was independently confirmed by the reviewers.

### PRISMA Flow Diagram

**Figure 1:**
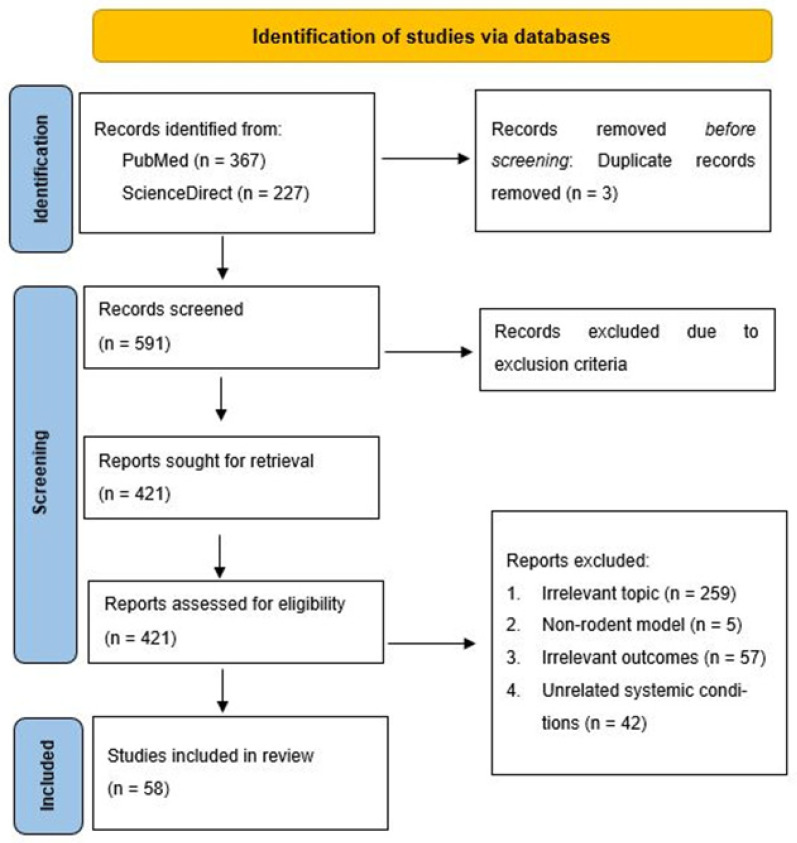
PRISMA flow diagram.

The entire selection process, from identification to inclusion, is illustrated in the PRISMA flow diagram (Figure 1). The diagram provides a detailed account of the number of records identified, screened, assessed for eligibility, and ultimately included in the review as well as the reasons for exclusion at each stage.

A total of 594 records were identified through database searches (PubMed and ScienceDirect), with 3 duplicates removed before screening. Of the 591 records screened, 421 full-text articles were sought for retrieval. Following eligibility assessment, 259 articles were excluded for reasons including irrelevant topics, 5 were excluded for non-rodent model studies, and 57 were excluded for irrelevant outcomes and unrelated systemic conditions (*n* = 42). The final review included 58 articles providing supplementary aggregated data.

## 3. Results

This study utilized PubMed and ScienceDirect as the primary databases for literature retrieval. A total of 594 records were initially identified, with 367 articles sourced from PubMed and 227 articles from ScienceDirect. After removing three duplicate records, 591 unique articles remained for further screening. The first phase involved screening the titles and abstracts to assess their relevance to the research objectives. Articles were excluded if they did not address the key focus of the review. Following this preliminary assessment, 421 articles were deemed potentially relevant and were sought for full-text retrieval. A full-text review was conducted on 421 studies, applying the predefined exclusion criteria. During this stage, 297 articles were excluded for the following reasons:

1. Irrelevant topic (*n* = 259)—Studies that did not align with the research scope.
2. Non-rodent model (*n* = 5)—Studies that used non-rodent models when rodent-based research was required.
3. Irrelevant outcomes (*n* = 57)—Studies that did not measure relevant clinical or biological outcomes.
4. Unrelated systemic conditions (*n* = 42)—Studies that focused on systemic diseases unrelated to the review’s scope.

After applying these criteria, a total of 58 studies were included in the systematic review. These studies comprised clinical trials, in vivo/in vitro experimental research, and mechanistic studies, contributing valuable insights into the relationship between periodontal disease and systemic conditions. This rigorous screening and selection process ensured that only methodologically sound and highly relevant studies were included, strengthening the evidence base for the review. See Figure 1 for the PRISMA flow diagram summarizing the study selection process.

### 3.1. Study Characteristics

The study characteristics and main findings of the included studies are summarized in Table 1, Table 2 and Table 3.

### 3.2. Results Regarding Prevalence of Periodontitis in Different Populations Around the World

In this systematic review, a total of eight studies [8,9,10,11,12,13,14,15] were included to assess the prevalence of periodontitis.

Main outcomes of the studies

According to the studies included in this review, the global prevalence of periodontitis ranges from 34% to 65%, with significant variations observed across different regions and populations. Epidemiological studies report prevalence rates of 39.9% to 55.5% in the United States, 52.8% to 69.3% across various age groups in China, 42.4% in India, 36% in Egypt, 34.6% in Thailand, 21.14% (severe cases) in Mexico, and 48.6% in Portugal. These disparities highlight the influence of demographic factors, geographic location, and methodological differences in study design. The observed variations underscore the complex interplay of socioeconomic conditions, access to dental care, cultural practices, and environmental factors in shaping the global burden of periodontitis. The main findings of the included studies are tabulated in Table 1.

### 3.3. Results Regarding Association Between the Prevalence of Cardiovascular Disease and Periodontal Diseases

The studies included in this review [16,17,18,19,20] revealed that, across diverse regions, there is a significant association between periodontal disease (PD) and cardiovascular disease (CVD).

Main outcomes of the studies

Epidemiological studies across diverse populations have consistently demonstrated a strong association between periodontal disease (PD) and cardiovascular disease (CVD). A large cross-sectional analysis conducted in the Netherlands involving 60,174 individuals revealed that those diagnosed with PD had more than twice the likelihood of developing CVD, emphasizing a significant epidemiological correlation. Similarly, a study in Romania reported that 75.5% of 147 patients diagnosed with CVD also had PD, further reinforcing this association. Longitudinal data from Sweden, based on a cohort of 856 older adults, indicated that PD was linked to an elevated risk of ischemic heart disease (IHD) and increased mortality, with the impact being particularly pronounced among women aged 60–93 years. In South Korea, a large-scale cross-sectional study involving 173,209 participants demonstrated a heightened prevalence of IHD among individuals with PD, supporting the notion that chronic periodontal inflammation contributes to systemic vascular pathology. Additionally, a cohort study from Thailand, which followed 1850 participants, found that severe PD significantly increased the risk of coronary heart disease (CHD), reporting a hazard ratio of 4.53. Collectively, these findings highlight the potential role of PD as a modifiable risk factor for CVD.

### 3.4. Results Regarding the Virulence Factors of Periodontal Bacteria and Their Cellular Signaling Pathway Contributing to Cardiovascular Disease

This systematic review includes eight human studies [27,28,29,30,31,32,33,34], 18 animal studies [24,26,35,36,37,38,39,40,41,42,43,44,45,46,47,48,49,50], and 20 in vitro studies [21,22,23,25,50,51,52,53,54,55,56,57,58,59,60,61,62,63,64,65] to investigate the molecular mechanisms underlying periodontal disease (PD) and cardiovascular disease (CVD). Notably, study [50] includes both animal and in vitro experiments and has been considered in both categories for analysis.

Main outcomes of the studies

Periodontal pathogens, particularly members of the Red Complex—*Porphyromonas gingivalis*, *Treponema denticola*, and *Tannerella forsythia*—along with *Fusobacterium nucleatum*, play a critical role in the pathogenesis of cardiovascular disease (CVD) through their distinct virulence factors and interactions with host cellular signaling pathways. *P. gingivalis* contributes to endothelial dysfunction, chronic inflammation, and atherogenesis by activating Toll-like receptors (TLR2 and TLR4), upregulating the NF-κB signaling pathway, and inducing the production of key pro-inflammatory cytokines, such as interleukin-6 (IL-6) and tumor necrosis factor-alpha (TNF-α). Similarly, *T. denticola* triggers immune responses through the TLR2/4-MyD88 pathways, leading to increased matrix metalloproteinase (MMP) activity, oxidative stress, and apoptosis, all of which contribute to vascular damage. *T. forsythia* exacerbates systemic inflammation and promotes atherogenesis by downregulating cholesterol transporters, including liver X receptors (LXRα and LXRβ) and ATP-binding cassette transporter A1 (ABCA1), resulting in elevated low-density lipoprotein (LDL) and C-reactive protein (CRP) levels. Furthermore, *F. nucleatum* disrupts endothelial barrier integrity through its adhesin FadA, which binds to vascular endothelial (VE)-cadherin, promoting endothelial permeability and facilitating bacterial dissemination. Additionally, *F. nucleatum* enhances foam cell formation and triggers pro-inflammatory responses via TLR-MyD88-NF-κB signaling, further contributing to vascular inflammation and atherosclerotic plaque development. These findings highlight the mechanistic link between periodontal pathogens and CVD. The virulence factors of periodontal pathogens and their molecular mechanism contributing to cardiovascular disease are summarized in Table 3.

### 3.5. Quality Assessment

The risk of bias and the quality assessment of the included studies were performed using the Grading of Recommendations, Assessment, Development, and Evaluations (GRADE) framework. The assessment summary is included in Supplement Table S1.

Risk of Bias: Assessment of study methodology.

Inconsistency: Variability in study results.

Indirectness: Relevance of evidence to the review question.

Imprecision: Uncertainty in effect estimates.

Publication Bias: Potential overrepresentation of positive findings.

No automation systems were used for this assessment. Manual evaluations were conducted to ensure objectivity and minimize bias. Discrepancies were resolved through discussion, with involvement from a third and fourth reviewer when necessary, to reinforce the reliability and validity of the assessments. Since the data obtained are heterogeneous, we adopted a narrative approach to synthesize and summarize the outcomes.

## 4. Discussion

### 4.1. Impact and Epidemiology of PD and CVD

The interaction between the oral commensal microbiome, its virulence factors, and the host immune response can trigger localized inflammation in the periodontium, which plays a significant role in the onset and progression of periodontal disease (PD) [66]. PD is a chronic inflammatory condition characterized by bacterial colonization and immune responses that affect the gingiva, periodontal ligament, and alveolar bone. This chronic inflammation is often initiated by accumulating a polymicrobial biofilm at or below the gingival margin, typically due to inadequate oral hygiene. This leads to a cycle of persistent immune activation and tissue damage. In advanced stages, prolonged inflammation can cause irreversible damage to these supporting structures, resulting in periodontal pocket formation, bleeding, and potential tooth loss due to tissue and bone destruction. PD not only damages gum tissues but also deteriorates the supporting bone, leading to tooth loss, pain, and compromised chewing function [67,68,69].

Periodontal disease (PD) is recognized as the sixth most prevalent disease worldwide, with a substantial global burden. Our review found that the prevalence of PD ranges from 34% to 65%, reflecting significant regional variations. For instance, studies report prevalence rates of 39.9–55.5% in the USA, 52.8–69.3% in China across different age groups, 42.4% in India, 36% in Egypt, 34.6% in Thailand, 21.14% in Mexico, and 48.6% in Portugal. These variations can be attributed to differences in demographic factors, oral hygiene practices, access to dental care, genetic predisposition, and study methodologies. Moreover, higher prevalence rates in certain populations suggest the need for targeted public health interventions and preventive strategies to mitigate the impact of PD and its associated systemic health risks. Further research is essential to standardize diagnostic criteria and assess the role of socioeconomic and environmental factors in PD prevalence globally [12,13,14,15,16].

The association between periodontal disease (PD) and cardiovascular disease (CVD) has been consistently demonstrated across diverse populations, reinforcing the growing evidence of an oral–systemic health link. The studies included in this review highlight significant correlations between PD and increased CVD risk. A large cross-sectional study in the Netherlands involving 60,174 individuals found that those with PD had more than twice the odds of developing CVD. Similarly, in Romania, a striking 75.5% of CVD patients exhibited PD, suggesting a strong epidemiological connection. Longitudinal data from Sweden further support this association, revealing that PD increases the risk of ischemic heart disease (IHD) and mortality, particularly among women aged 60–93 years. In South Korea, a nationwide cross-sectional study of 173,209 participants identified a higher prevalence of IHD in individuals with PD, reinforcing the systemic impact of oral inflammation. Additionally, a Thai cohort study demonstrated a significant relationship between severe PD and coronary heart disease (CHD), with a hazard ratio of 4.53, underscoring the potential role of periodontal inflammation in cardiovascular pathology [16,17,18,19,20]. These findings collectively suggest that PD may serve as a contributing factor in CVD development. The underlying mechanisms likely involve systemic inflammation, bacterial dissemination, and immune dysregulation, all of which contribute to endothelial dysfunction and atherogenesis.

### 4.2. Cytokine, Chemokine, and Bacterial Metabolites in PD

The host’s immune response plays a critical role in determining immune fitness, which can maintain normal tolerance and homeostasis within the dental biofilm. The initial step of host immune response to local dysbiotic microbes involves direct interactions between the microbiome and host cells, which include periodontal tissue cells such as mucosal epithelial cells, gingival fibroblasts, and local immune cells [70]. Gram-negative periodontal pathogenic bacteria contain endotoxins and lipopolysaccharides (LPS) on their outer membrane and evoke the local immune response. When the bacterial LPS interact with the gingival tissue, it initiates inflammation and tissue breakdown, progressing tooth mobility. Continuous stimulation and damage caused by the microbiome and mechanical forces like mastication recruit immune cells, including mononuclear phagocytes (MNPs), antigen-presenting cells (APCs), and specific T-cell subsets such as Th17 cells [71]. The inflammatory response increases gingival crevicular fluid, which contains breakdown products of collagen and immune factors, such as immunoglobulins, complement proteins, cytokines, chemokines, and remnants of immune cells like polymorphonuclear leukocytes. Activation of polymorphonuclear cells results in degranulation, chemotaxis, phagocytosis, and the release of reactive oxygen species (ROS) [72,73]. Additionally, epithelial cells and collagen peptides are degraded by matrix metalloproteinases (MMPs) [74]. Studies show that the subgingival microbiomes in periodontitis have revealed upregulated expression of genes responsible for proteolytic enzymes, iron acquisition, and lipopolysaccharide synthesis [27]. These interactions initiate the first wave of cytokine secretion, driven by the activation of pattern recognition receptors and their downstream signaling pathways. Key cytokines involved in this phase include the IL-1 family, IL-6 family, and tumor necrosis factor (TNF), all of which play crucial roles in promoting inflammation and tissue destruction [71,75]. In the later stage, additional cytokines, which are closely linked to the differentiation of specific lymphocyte subsets, are secreted by MNPs, APCs, and local lymphocytes in response to microbial stimulation. Cytokines from the IL-1 and IL-6 families, among others, activate signaling pathways that drive the maturation and differentiation of immune cells [76].

Several pro-inflammatory cytokines, including IL-1, IL-6, IL-12, IL-17, IL-18, IL-21, TNF-α, and IFN-γ, play a critical role in the pathogenesis of periodontitis. Elevated levels of IL-6 have been consistently observed in the gingival crevicular fluid (GCF) and gingival tissues of periodontitis patients compared to healthy individuals [77]. This heightened expression of IL-6 correlates with disease severity, while a reduction in systemic IL-6 levels is seen following periodontal treatment, aligning with clinical improvements in periodontal health [28,29,30]. IL-1 and TNF-α are key cytokines that play a significant role in amplifying the inflammatory response at the cellular level by inducing the production of other inflammatory mediators, such as IL-6, IL-8, MMPs, and prostaglandin E2 (PGE2). These mediators contribute to tissue degradation and inflammation in PD. Experimental studies in animal models have confirmed the pathogenic role of cytokines such as IL-1 and TNF-α, which exacerbate periodontal tissue damage and play a key role in the progression of the disease [78].

Chemokines play a key role in driving chemotaxis, guiding immune cells to sites of inflammation. In periodontitis, chemokines, such as IL-8, monocyte chemoattractant protein-1 (MCP-1), and macrophage inflammatory protein-1α (MIP1α), are pivotal in attracting neutrophils and other leukocytes to the affected tissue. IL-8, produced by a variety of cells, including monocytes, lymphocytes, epithelial cells, endothelial cells, and fibroblasts, is secreted in response to inflammatory signals, like IL-1, TNFα, and lipopolysaccharides (LPS). MCP-1, similarly, produced by endothelial cells, epithelial cells, and fibroblasts, is triggered by bacterial components and inflammatory mediators [79]. Studies show that the levels of MCP-1, MIP1α, and RANTES (Regulated on Activation, Normal T cell Expressed and Secreted) are elevated in gingival biopsies and gingival crevicular fluid (GCF) of periodontitis patients. Interestingly, periodontal treatment can reduce chemokine levels in GCF, highlighting their involvement in disease progression and response to therapy. Arachidonic acid metabolites, including prostaglandins, leukotrienes, MMPs, and TIMPs, play a significant role in the pathogenesis of periodontitis, a chronic inflammatory disease affecting the tissues surrounding the teeth. These metabolites, including prostaglandins and leukotrienes, are derived from arachidonic acid and exert various biological effects. Prostaglandins, particularly PGE2, are key inflammatory mediators in periodontitis. They stimulate inflammatory responses, promote tissue destruction, and contribute to bone resorption. Leukotrienes, while primarily associated with asthma and allergies, also play a role in periodontitis. Leukotriene B4 (LTB4) has been implicated in the disease progression, while Resolvin E1 (RvE1) exhibits anti-inflammatory effects and may be beneficial in treating periodontitis. MMPs and their inhibitors (TIMPs) are involved in tissue breakdown and repair. Increased MMP activity and decreased TIMP levels are observed in periodontitis, contributing to tissue destruction.

Reactive oxygen species (ROS) have been implicated as key mediators in the pathogenesis of PD [80]. Excessive ROS production, leading to oxidative stress, has been shown to damage various biomolecules, including lipids, proteins, and DNA. This oxidative stress triggers a cascade of pro-inflammatory events, including osteoclast activation that ultimately results in bone loss. Furthermore, ROS downregulation of nuclear factor erythroid 2-related factor 2 (Nrf2), a critical antioxidant regulator, contributes to the progression of PD [81]. In patients with chronic periodontitis, Nrf2 is downregulated in neutrophils, leading to a cellular redox imbalance. Studies show that upregulating Nrf2 reduces oxidative stress in periodontitis models [18,35,36].

### 4.3. PD and Systemic Health

PD extends beyond the confines of the oral cavity, impacting systemic health both directly and indirectly through mechanisms involving chronic inflammation and maladaptive immune response to periodontal pathogens and their by-products, as described above. Directly, the highly vascularized periodontal environment allows for the oral microbiome to trigger transient bacteremia during routine activities like chewing and brushing, especially in cases of microbial dysbiosis. Periodontal pathogens such as *P. gingivalis* can penetrate compromised gingival barriers, enter the bloodstream, and disseminate to distant organs, causing systemic inflammation, and such bacteria have been detected in thrombi from patients with acute myocardial infarction, suggesting a role in atheromatous plaque pathology [31,51]. *P. gingivalis* also weakens epithelial integrity by disrupting grainy head-like 2 and connexin expression, facilitating bacteremia [51]. Indirectly, the chronic inflammation caused by PD can contribute to systemic inflammatory conditions [82]. Established associations include respiratory diseases, cardiovascular diseases, rheumatoid arthritis, obesity, and diabetes, with oral manifestations also observed in Crohn’s disease [36]. Recent investigations have revealed a potential role of PD in exacerbating or even driving systemic conditions such as Alzheimer’s disease and cancer [83]. Research shows that poor oral hygiene and periodontitis are linked to increased respiratory diseases, including pneumonia and chronic obstructive pulmonary disease (COPD). Improved oral care can reduce these risks, especially in high-risk elderly populations. Mechanisms include aspiration of oral bacteria and changes in saliva and respiratory epithelium due to periodontal inflammation. While numerous systemic diseases are associated with periodontal disease, this review specifically focuses on its connections with cardiovascular disease.

### 4.4. PD and CVD Inflammatory Axis

Cardiovascular disease (CVD) is a broad term encompassing conditions that affect the arteries and blood vessels throughout the body, primarily including coronary heart disease, stroke, and peripheral vascular disease [84]. Coronary heart disease (CHD) is characterized by the accumulation of atherosclerotic plaques within the coronary arteries, leading to arterial narrowing and potentially resulting in conditions such as myocardial ischemia and infarction [85]. CHD remains a leading cause of mortality worldwide, accounting for nearly seven million deaths and 129 million disabilities each year [86]. This is likely due to systemic inflammation caused by a periodontal infection [32]. While vascular disease is undoubtedly multifactorial, substantial evidence shows that chronic vascular infections and persistent systemic inflammation are significant risk factors [87,88]. Numerous epidemiological and clinical studies have demonstrated a significantly higher risk of atherosclerosis (AS) among patients with periodontitis, with risk estimates reaching up to a 95% confidence interval [17,20,89,90]. The prevalence of cardiovascular disease in patients with periodontitis is depicted in Table 2. The evidence indicates that individuals with periodontal disease have a 34% higher risk of developing heart-related conditions, highlighting the strong association between periodontal health and cardiovascular disease [91]. Although the clinical association between periodontal pathogens and AS is unclear, growing evidence in laboratory animal experiments has confirmed the promotional role of red complex bacteria and others in the progress of AS and endothelial dysfunction [37,38,39,40]. AS is the buildup of fats and other substances in and on the medium- and large-sized artery walls regarded as the pathogenic basis of CVD. The buildup is called plaque, which can eventually restrict blood flow to important organs causing stroke, myocardial infarction, or acute coronary syndrome.

**Figure 2:**
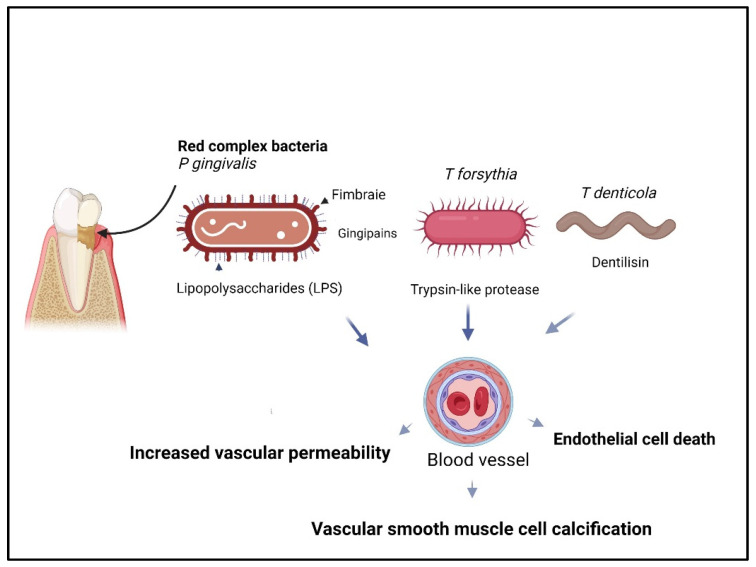
Role of red complex bacteria in atherosclerosis. *Porphyromonas gingivalis*, *Tannerella forsythia*, and *Treponema denticola* contribute to atherosclerosis through virulence factors such as gingipains, lipopolysaccharides, fimbriae, trypsin-like proteases, and dentilisin. These factors enhance vascular permeability, promote inflammatory cell and lipid infiltration, induce endothelial dysfunction via endotoxins and proteases, and drive vascular smooth muscle cell calcification. Together, these processes accelerate plaque formation and atherosclerosis progression (the figure is created in BioRender.com).

Periodontal pathogens, including red complex bacteria, *Aggregatibacter actinomycetemcomitans*, *Prevotella intermedia*, *Streptococcus mutans*, and *Streptococcus sanguinis*, have been detected in atherosclerotic (AS) plaque lesions and are commonly associated with CVD [33,41,92]. These pathogens have been shown to increase the risk and progression of AS, suggesting that infection by periodontal bacteria contributes to both the formation and progression of AS plaques [34]. Oral bacteria and/or their DNA are commonly discovered in heart valves, and atherosclerotic plaque can induce epigenetic alterations, causing dysregulated signaling pathways related to host immune responses, cell adhesion, migration, and proliferation [52,93]. Similarly, antibodies produced against periodontal pathogens can cross-react with antigens in cardiovascular tissues, potentially exacerbating inflammation and promoting atherosclerotic plaque formation [42,53,94,95,96,97]. For instance, heat shock proteins from periodontal pathogens can trigger antibodies that cross-react with human heat shock proteins, leading to increased cytokine production and activation of monocytes and endothelial cells. Additionally, antibodies against malondialdehyde-humanized-modified LDL (MAA-LDL) and oxidized LDL (OxLDL) have been associated with AS (Figure 2).

As the atherosclerosis progresses, endothelial damage initiates platelet adhesion and activates endothelial cells (EC), promoting inflammation and the recruitment of monocytes. These recruited monocytes differentiate into foam cells, thereby contributing to plaque formation. Cytokines produced by monocytes, including tumor necrosis factor-alpha (TNF-α) and interleukins (IL-1, IL-6, and IL-8), are released in response to various stimuli associated with periodontal infection. Among these stimuli, endotoxins such as lipopolysaccharides (LPS) released by periodontal pathogens exacerbate the inflammatory process by upregulating adhesion molecules like intercellular adhesion molecule-1 (ICAM-1) and facilitating interactions between macrophage migration inhibitory factor and the receptors CD74 and CXCR4, which further promote the development of atherosclerotic plaques [54,55]. Additionally, red complex bacteria enhance the production of pro-inflammatory cytokines and the vasoconstrictor angiotensin II in endothelial cells [56]. A meta-analysis conducted by Joshi et al. (2021) found that elevated levels of IgG antibodies targeting *P. gingivalis* and *A. actinomycetemcomitans* are modestly linked to a higher risk of coronary heart disease [98]. An animal model study demonstrated that mice orally infected with *T. denticola* exhibited enlarged arterial plaques, reduced serum nitric oxide (NO) levels, and elevated serum levels of very-low-density lipoprotein (VLDL) and oxidized low-density lipoprotein (ox-LDL) [24]. In a cross-sectional study, higher triglyceride (TG) levels and lower high-density lipoprotein (HDL) levels were significantly associated with the presence of *T. denticola* in individuals with chronic periodontitis [54]. The pathological progression of atherosclerotic plaque involves vascular smooth muscle cells (VSMCs), which respond to lipid infiltration and arterial injury by contributing to intimal thickening and foam cell formation.

**Figure 3:**
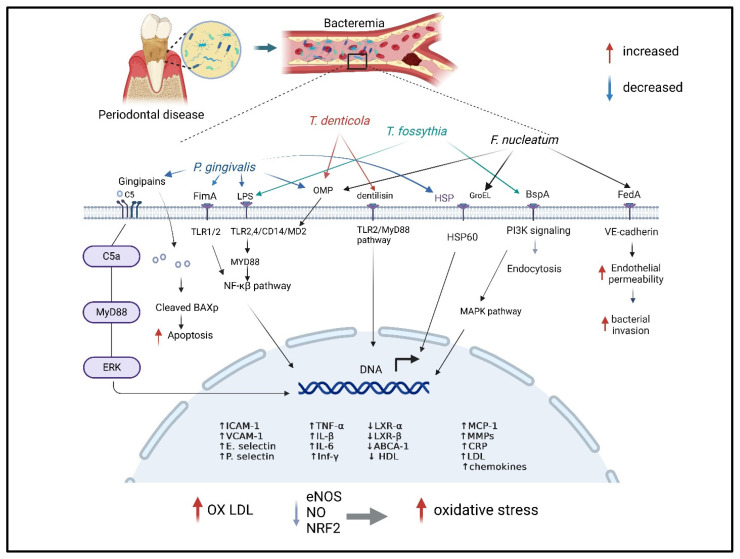
Mechanisms of acute inflammation and molecular pathways induced by red complex bacteria and *Fusobacterium nucleatum* in endothelial cells. This figure depicts the interactions between *Porphyromonas gingivalis*, *Tannerella forsythia*, *Treponema denticola*, *Fusobacterium nucleatum*, and endothelial cells, highlighting their molecular mechanisms. The bacteria attach to endothelial cells via specific receptors and release virulence factors that activate pathways such as TLR-NF-κB, MAPK, MyD88, and PI3K signaling. These activations result in the upregulation of intercellular adhesion molecule-1 (ICAM-1), vascular cell adhesion molecule-1 (VCAM-1), E-selectin, P-selectin, and inflammatory cytokines, leading to enhanced adhesion molecule expression and chemokine secretion. Degradation of ApoB-100, aggregation of ox-LDL, and foam cell generation contribute to atherosclerosis. Reactive oxygen species (ROS) production, antioxidant depletion, reduced nitric oxide levels, and cardiomyocyte apoptosis via BAX protein activation further exacerbate endothelial dysfunction. Upregulation of matrix metalloproteinases (MMPs) and pro-inflammatory cytokines promotes vascular damage, linking these mechanisms to the progression of atherosclerosis (the figure is created in BioRender.com).

*Porphyromonas gingivalis*, a key bacterium within the red complex group associated with periodontal disease, is also linked to cardiovascular disease (CVD). The pathogenic impact of *P. gingivalis* on CVD is driven by a sophisticated arsenal of virulence factors that disrupt host cellular signaling pathways, induce chronic inflammation, and facilitate immune evasion (Figure 3). The fimbriae (FimA) play a central role in promoting bacterial motility, biofilm formation, and the adhesion and invasion of endothelial cells, leading to vascular activation and dysfunction [99]. Lipopolysaccharides (LPS) from *P. gingivalis* activate host immune receptors, specifically Toll-like receptors TLR2 and TLR4, which, in turn, upregulate intercellular adhesion molecules (ICAM-1, VCAM-1) and selectins (E- and P-selectin). This response increases cytokine release, particularly IL-6, and promotes ECM-receptor interaction, amplifying immune cell recruitment to sites of infection and enhancing systemic inflammatory responses [99].

A hallmark of *P. gingivalis* pathogenicity is the secretion of gingipains, a family of cysteine proteases that play multiple roles in host–pathogen interactions. Gingipains activate the NF-κB pathway, leading to the upregulation of pro-inflammatory cytokines and the degradation of extracellular matrix (ECM) components, including integrin–fibronectin-binding factors, cytokines, immunoglobulins, and complement proteins [99]. This proteolytic activity weakens tissue barriers and propagates inflammation [57]. Additionally, gingipains contribute to atherosclerosis by degrading ApoB-100, facilitating LDL aggregation, foam cell formation, and increased lipid deposition in the vascular endothelium. In cardiomyocytes, gingipains induce apoptosis via activating the pro-apoptotic bax protein through cleavage at the Arg34 site, linking *P. gingivalis* to cardiovascular pathologies.

Other virulence factors enhance *P. gingivalis* survival and dissemination. Hemolysin and hemagglutinin proteins agglutinate and lyse erythrocytes, securing an iron source crucial for bacterial metabolism [100]. The capsule surrounding *P. gingivalis* confers protection from host immune responses, enhancing intracellular survival and increasing virulence [99]. Outer membrane vesicles (OMVs) produced by *P. gingivalis* further facilitate host cell invasion, immune evasion, and antibiotic resistance through mechanisms that involve disruption of host immune defenses and cellular destruction [101,102]. Additionally, a study showed *P. gingivalis*-derived gingipains induce the proliferation of rat aortic smooth muscle cells. Virulence factors from *P. gingivalis* further drive VSMC calcification through mechanisms involving the upregulation of osteogenic genes and signaling pathways, such as the runt-related transcription factor 2 and extracellular-regulated kinase signaling [58,59]. A common pathogenic outcome of these virulence factors includes increased intracellular reactive oxygen species (ROS) production, depletion of cellular antioxidants, and reduction in nitric oxide (NO) bioavailability, all of which drive oxidative stress. This oxidative imbalance along with the activation of the NF-κB pathway and upregulation of IL-1β and TNF-α promote endothelial cell apoptosis and death, further contributing to vascular dysfunction.

*Treponema denticola*, a key member of the red complex periodontal pathogens, significantly contributes to endothelial dysfunction and cardiovascular disease (CVD) through various virulence mechanisms, impacting host cellular signaling and immune responses. This bacterium is equipped with multiple virulence factors, including the major outer sheath protein (MSP), dentilisin, acylated chymotrypsin-like protease complex (CTLP), and lipooligosaccharide, each playing a distinct role in pathogenesis (Figure 3). MSP facilitates bacterial adherence to host extracellular matrix components, such as fibronectin, plasminogen, and laminin, promoting bacterial aggregation and invasion. MSP can activate innate immunity through TLR2, leading to inflammatory responses. MSP’s proteolytic enzymes further contribute to immune modulation by degrading host proteins, weakening host defense mechanisms. Dentilisin, a prominent virulence factor, enhances *T. denticola* pathogenicity by activating complement component C3. Dentilisin also hydrolyzes key inflammatory cytokines, including IL-1β, IL-6, and TNF-α, and the immunoglobulins IgG and IgA. This degradation disrupts intercellular signaling and host matrix integrity, further aggravating disease progression. Activation of the TLR2 and TLR4 pathways by dentilisin leads to the upregulation of MyD88-dependent signaling, which triggers pro-inflammatory cytokines and excessive endothelial cell activation, contributing to endothelial dysfunction and CVD pathogenesis. The lipooligosaccharide of *T. denticola* also stimulates TLR4 signaling, inducing the release of TNF-α, nitric oxide, and other pro-inflammatory cytokines from immune cells. This activation further amplifies the inflammatory cascade in endothelial cells, promoting vasoconstriction, cell apoptosis, and adhesion molecule expression, which are critical factors in endothelial damage and atherosclerosis. Additionally, *T. denticola* metabolites, including short-chain fatty acids (SCFAs), ammonia, reactive oxygen species (ROS), and bioactive lipids, contribute to inflammation and oxidative stress, exacerbating cardiovascular risk [2,103]. Collectively, these virulence factors from *T. denticola* form a synergistic assault on endothelial cells, modulating immune responses, degrading host structural proteins, and inducing chronic inflammation and oxidative stress. This cumulative impact underscores the significant role of *T. denticola* in periodontal disease progression and its associated risks for endothelial dysfunction and cardiovascular disease.

*T. forsythia*, the third and well-established key member of the red complex bacteria linked to periodontal disease, has shown increasing associations with cardiovascular disease (CVD). Several studies have highlighted a strong connection between *T. forsythia* and atherosclerosis. A recent meta-analysis reported a prevalence of *T. forsythia* in 43.7% of atherosclerotic plaques in coronary arteries [104]. Similarly, another study found that patients with coronary artery disease had elevated IgA levels against *T. forsythia* in saliva samples compared to healthy individuals [105]. Animal models of atherosclerosis (AS) further demonstrate the role of *T. forsythia* and its protein BspA in promoting plaque enlargement, increasing serum CRP and LDL levels, and decreasing HDL levels [43,105]. Additionally, *T. forsythia* reduced liver expression of key lipid-regulatory proteins, such as LXRα, LXRβ, and ABCA1, which are crucial for cholesterol homeostasis [26].

The virulence factors of *T. forsythia* contribute significantly to its pathogenicity (Figure 3). The S-layer proteins enable bacterial adhesion and invasion by interacting with lectin-like receptors on host cells and coaggregating bacteria like *F. nucleatum*. Surface lipoproteins from *T. forsythia* stimulate the release of pro-inflammatory cytokines and apoptosis in host cells. These lipoproteins activate the transcription factor NF-κB through TLR2-mediated signaling, leading to cytokine production. BspA, a major protein of *T. forsythia*, facilitates bacterial adherence and invasion of endothelial cells, induces foam cell formation, and contributes to the progression of atherosclerotic lesions [26]. Studies have shown that BspA-mediated coaggregation with *T. denticola* and *F. nucleatum* exacerbates foam cell formation in THP-1 cells and accelerates atherosclerosis progression in ApoE (^−/−^) mice [45]. Furthermore, *T. forsythia* and BspA elevate serum CRP and LDL levels while decreasing HDL, further promoting atherosclerotic development [43]. The role of *T. forsythia* in promoting atherosclerosis (AS) remains partially understood; however, its various virulence factors contribute significantly to the inflammatory processes and lipid dysregulation that drive atherosclerosis. By evading innate immune responses, promoting chronic inflammation, and facilitating foam cell formation, *T. forsythia* plays a key role in the progression of atherosclerosis, thereby linking periodontal disease to increased cardiovascular risk.

*F. nucleatum*, a keystone pathogen linked with red complex bacteria, plays a significant role in periodontitis progression and has recently garnered attention for its association with atherosclerosis [45]. Animal studies highlight several pathogenic mechanisms by which *F. nucleatum* may exacerbate atherosclerosis, including its high invasiveness, activation of innate immunity, induction of dyslipidemia, and promotion of systemic inflammation [46,47]. The pathogen influences macrophage polarization, skewing cells toward a pro-inflammatory M1 phenotype, which contributes to foam cell formation, cell apoptosis, and extracellular matrix degradation through matrix metalloproteinases, ultimately accelerating plaque formation and rupture [44,48,60,106]. Further animal model research reveals that intraperitoneal injection of heat-killed *F. nucleatum* and its heat shock protein GroEL can lead to endothelial dysfunction and advance atherosclerotic lesion progression, largely by triggering autoimmune responses. In vitro studies validate these findings, demonstrating that *F. nucleatum* can activate macrophage PI3K-AKT/MAPK/NF-κB signaling pathways, which intensify inflammatory responses, increase cholesterol uptake, inhibit lipid excretion, and promote lipid accumulation [61]. Additionally, *F. nucleatum* infection has been shown to decrease the expression of platelet endothelial cell adhesion molecule-1 (PECAM-1) and induce endothelial cell death, further contributing to vascular pathology and the development of atherosclerosis [49]. The virulence factors of *F. nucleatum* further clarify its role in promoting endothelial dysfunction and atherosclerosis. Notably, the *F. nucleatum* adhesion protein FadA binds to VE-cadherin on endothelial cells, increasing vascular permeability, disrupting normal cell function, and impairing vascularization (Figure 3). This binding facilitates bacterial invasion and induces inflammation within endothelial tissues, intensifying vascular damage [62].

The bacterial heat shock protein GroEL also plays a pivotal role in atherosclerosis progression [50]. It has been associated with decreased serum HDL levels; elevated serum C-reactive protein (CRP), interleukin-6 (IL-6), and LDL levels; and foam cell formation, all of which are critical risk factors for atherosclerotic development. GroEL induces a pro-inflammatory response by activating the TLR-MyD88-NF-κB signaling pathway in endothelial cells, leading to the upregulation of pro-inflammatory molecules such as MCP-1 and IL-8 as well as adhesion molecules like ICAM-1 and VCAM-1. This activation promotes the recruitment of immune cells, perpetuating local inflammation and contributing to plaque formation [62]. Additionally, GroEL presents cross-reactive potential, where antibodies against bacterial GroEL may also bind to human heat shock protein 60 (hHSP60) on endothelial cells [44]. This antibody cross-reactivity can induce endothelial dysfunction, further escalating the inflammatory response and advancing atherosclerosis. Importantly, it has been shown that *F. nucleatum* alone cannot induce atherosclerosis; rather, it requires the physical, metabolic, and nutritional presence of other periodontal bacteria to act synergistically, thereby amplifying its pathogenic effects [48]. In the polymicrobial environment of periodontal disease, *F. nucleatum* interacts closely with red complex bacteria, such as *P. gingivalis* and *T. denticola*, creating a biofilm that enhances the survival, virulence, and pathogenic potential of each organism [45]. This cooperative biofilm formation contributes to the persistence of inflammation and increases the bacterial load, both critical for disease progression. Within this cooperative network, *F. nucleatum* and its virulence factors, like FadA and GroEL, significantly increase endothelial permeability, initiate immune responses, and induce systemic inflammatory responses, all implicated in the development of atherosclerotic lesions [107]. This multifactorial interplay among periodontal pathogens emphasizes the complex microbial dynamics involved in cardiovascular risk associated with periodontal disease. The virulence factors, their pathogenicity, and the cellular signaling pathway leading to CVD progress are tabulated in Table 3.

### 4.5. Periodontal Pathogens and Endothelial Oxidative Stress

Lipopolysaccharides (LPS) from periodontal pathogens may activate Toll-like receptors (TLRs) and other pattern-recognition receptors in vascular endothelial cells, inducing inflammation and oxidative stress [45]. Endothelial oxidative stress promotes cytokine release and monocyte adhesion, leading to endothelial dysfunction, which is, as described previously, a precursor to atherosclerotic lesions [108]. Research has proven that oral pathogenic bacteria induce severe endothelial oxidative stress by significantly increasing the output of total superoxide free radicals and ROS. This process is mainly prompted by the TLRs–Nuclear factor kappa B (NF-κB) signaling pathway [63]. TLRs mediate the recognition of bacterial endotoxins, activate the downstream signaling pathway NF-κB and its active subunit p65, and subsequently trigger oxidative stress. Subsequently, interleukin-1β (IL-1β) and IL-18 begin to be secreted, triggering further oxidative stress and inflammatory processes in the endothelium and destruction of vascular endothelial homeostasis mainly by inhibiting the proliferation and apoptosis of endothelial cells. Nitric oxide is critical in preventing oxidative stress and maintaining homeostasis by inhibiting the production of ROS. For instance, *P. gingivalis* leads to nitrifying stress and impaired endothelial function by upregulating inducible nitric oxide synthase (iNOS), downregulating endothelial nitric oxide synthase (eNOS), and regulating the release of NO in EC via change in the glycogen synthase kinase-3 (GSK-3β)/tetrahydrobiopterin (BH4)/eNOS/nuclear factor erythroid-derived 2-like 2 (Nrf2) pathways [109]. In previous animal model studies, polybacterial infections significantly decrease serum NO levels and the bioavailability of BH4 due to the inhibition of the expression of dihydrofolatereductase (DHFR) that predominates the conversion of BH2 to BH4 and the rate-limiting enzyme GTP cyclohydrolase 1 (GCH-1) responsible for the biosynthesis of BH4 [109,110]. NrF2 can protect cells from oxidation by activating antioxidant enzymes, including GSH synthase (GCSc, GCSm) and heme oxygenase-1, which is essential for cell protection. In the vascular tissues of mice infected with *P. gingivalis*, from previous studies, the level of NrF2 was significantly reduced [63]. After promoting oxidative stress, the pathogen triggers an inflammatory response in vasculature. IL-1β, IL-6, TNFα, and interferon-gamma (IFN-γ), as pro-inflammatory factors, become increased in the EC, which enhances monocyte migration and adhesion and, thereby, promotes the development of AS [64]. The heat shock protein 60 (HSP60) of oral pathogenic bacteria is incredibly immunogenic and induces apoptosis of human umbilical vein endothelial cells via suppressing the expression of eNOS and vascular endothelial cadherin [65]. The apoptosis of endothelial cells facilitates the proliferation and migration of smooth muscle cells, which in turn promotes blood coagulation and leukocyte infiltration, ultimately resulting in endothelial dysfunction.

### 4.6. Inflammatory Biomarkers in Periodontitis and Cardiovascular Disease

C-reactive protein (CRP), an acute-phase reactant produced primarily in the liver in response to inflammatory cytokines like IL-6, is elevated in both CVD and periodontitis. Elevated CRP levels are linked to atheroma formation by activating the complement system and contributing to inflammatory responses. Additionally, chronic periodontitis is associated with elevated systemic levels of other pro-inflammatory mediators, such as fibrinogen, haptoglobin, and IL-18, alongside decreased anti-inflammatory markers like IL-4. Despite accounting for confounding factors such as smoking, studies consistently show significant associations between serum levels of CRP and IL-6 and periodontitis.

Animal studies on CVD have shown that periodontitis and bacterial infections increase inflammatory serum markers. In non-human primates, ligature-induced periodontitis elevated acute-phase proteins, such as CRP and fibrinogen, which normalized after treatment. Studies showed that *A. actinomycetemcomitans* increased serum markers (IL-6, IL-8, TNF-α, MCP-1) and atherosclerotic plaque size. Similarly, *P. gingivalis* infusion in mice caused myocardial infarction or myocarditis, with inflammation dependent on IL-17A, suggesting Th17 pathways play a role in *P. gingivalis*-induced cardiovascular inflammation. MMPs are implicated in both periodontal tissue destruction and CVD due to their role in atherosclerotic plaque rupture, which can be triggered by oral bacterial products. *P. gingivalis* proteases (gingipains) are proposed to stimulate MMP production and activate latent MMPs, potentially linking periodontitis to CVD. Although fewer studies have focused on MMPs compared to other mediators, evidence shows decreased serum MMP-9 levels following periodontal treatment and associations between elevated MMP-9/TIMP-1 levels in periodontitis patients and increased carotid intima-media thickness, highlighting a possible connection. Elevated fibrinogen, a marker of systemic inflammation, increases the risk of AS by promoting endothelial activation and platelet aggregation. It stimulates the production of pro-inflammatory cytokines and is found in atheromas. Studies show that patients with periodontitis have higher plasma fibrinogen levels, and the number of periodontal pockets correlates with fibrinogen levels, linking periodontitis to CVD risk.

## 5. Conclusions

Periodontal disease (PD) is a multifactorial inflammatory condition with a global prevalence ranging from 34% to 65%, exhibiting significant regional variations influenced by demographic, socioeconomic, and environmental factors. Driven by dysbiotic microbial communities, dysregulated immune responses, and systemic inflammatory and oxidative pathways, PD contributes to cardiovascular disease (CVD), diabetes, and other chronic disorders. Key pathogens, such as *P. gingivalis*, *T. denticola*, *T. forsythia*, and *F. nucleatum*, exacerbate systemic inflammation through mechanisms involving Toll-like receptor (TLR) activation, NF-κB signaling, and nitric oxide dysregulation, promoting endothelial dysfunction, oxidative stress, and atherogenesis. Elevated systemic biomarkers, including CRP, interleukins, and MMPs, further underscore the link between PD and vascular pathology.

## 6. Limitations and Future Directions

This systematic review has limitations, including potential exclusion of non-English studies, access to full-text articles and regional journals, and insufficient representation of low- and middle-income socioeconomic conditions. Variability in study designs and exclusion of gray literature introduced heterogeneity, challenging consistency. Despite strong epidemiological and mechanistic evidence, further research is needed to fully elucidate the molecular pathways and clinical implications of periodontal disease’s (PD) systemic impact. Future research should focus on modulating inflammatory mediators (e.g., IL-1, IL-6, TNF-α, ROS) and enhancing protective pathways (e.g., Nrf2, Resolvin E1). Advancing the understanding of host–microbiome interactions and identifying novel biomarkers are critical for early diagnosis and management. Employing multi-omics profiling, high-throughput sequencing, and bioinformatics can elucidate microbial–host dynamics and genetic/epigenetic modifications. Longitudinal and interventional studies are needed to evaluate periodontal treatment’s impact on systemic outcomes.

## Figures and Tables

**Table 1:** Summary of characteristics of included studies regarding the prevalence of periodontitis in different populations around the world.

Country of Study	Study Design	Sample Population	Key Findings	References
USA	A cross-sectional study	9034 adults aged 30 years or older who underwent a periodontal examination from the National Health and Nutrition Examination Survey from 2011 to 2014	The prevalence of periodontitis in this population was 39.9%.	[8]
USA	A retrospective analysis	1131 patients	Overall periodontitis prevalence for this population was 55.5%.	[9]
China	An observational study	Three age groups: the first being 35–44 years old, the second group being ages 55 to 64 years, and the final group being 65 to 74 years old	The frequency of subjects with periodontitis was 52.8%, 69.3%, and 64.6% in the three age groups, respectively.	[10]
Egypt	A cross-sectional study	314 patients aged from 19 to 39 years	Prevalence of periodontitis was 36% in this study.	[11]
India	An observational study	A total of 500 adults aged between 30 and 60 years	Periodontitis was observed in 42.4% of this population.	[12]
Mexico	A cross-sectional study	440 people over 15 years of age	A total of 21.14% of the participants showed severe periodontitis and required periodontal intervention.	[13]
Portugal	A cross-sectional study	941 patients	Periodontitis prevalence for this study was 48.6%.	[14]
Thailand	A cross-sectional study	2086 participants	An observation of periodontitis was seen in 34.6% of the participants.	[15]

**Table 2:** Summary of characteristics of included studies regarding association between the prevalence of cardiovascular disease and periodontal diseases.

Country/Region of Study	Study Design	Disease Stage	Sample Size	Key Findings	References
Netherlands	Cross-sectional analysis conducted using data extracted from the electronic health records of individuals registered at the ACTA	Patients hospitalized due to various conditions dealing with the CV system	60,174 individuals	Out of 60,174 individuals, 9730 had PD, and 455 had PD with CVD. Individuals with PD have more than twice the odds of having CVD.	[16]
Romania	Oral health examination is conducted in patients at the Emergency Hospital of Sibiu	Patients were hospitalized due to some CVD with dental health assessment	221 patients aged between 46 and 76 years	Out of 147 patients with cardiovascular disease, 75.5% had PD.	[17]
Karlskrona, Sweden	A prospective longitudinal cohort study, observing older individuals’ relationship between PD and CVD/death	Patients were hospitalized or previously hospitalized due to CVD or stroke, and then dental records were reviewed	856 (primary sample size was 858) individuals aged 60 years and above	Significant association between PD and the incidence of ischemic HD especially in women aged between 60 and 93 years. PD poses a significant risk factor for the development of ischemic HD and mortality in older adults.	[18]
South Korea	Cross-sectional study on epidemiological data	Focused on patients with ischemic heart disease	173,209 participants with 9973 participants reporting PD	Participants with PD had a significantly higher risk of ischemic heart disease compared to those without PD.	[19]
Thailand	Observational cohort study	Variations of mild, moderate, and severe over the follow-up period	1850 participants with an age of 47–73 years	Participants with severe PD had a significantly higher risk of developing CHD compared to those with no/mild to moderate PD, with a hazard ratio of 4.53.	[20]

**Table 3:** Summary of characteristics of included studies regarding the virulence factors of periodontal (Red Complex) bacteria and their cellular signaling pathway contributing to cardiovascular disease.

Bacteria	Virulence Factors	Pathogenicity and Cellular Signaling Pathway	References
*Porphyromonas gingivalis*	Fimbriae (FimA), Lipopolysaccharide (LPS), Gingipains, Hemolysin and hemagglutinin, Capsule, Outer membrane vesicles (OMVs)	Bacterial motility, biofilm formation, endothelial cell adhesion/invasion, activation, and dysfunction. TLR2 and TLR4 activation; upregulation of intercellular adhesion molecule-1 (ICAM-1), vascular cell adhesion molecule-1 (VCAM-1), E-selectin, and P-selectin; increase in serum IL-6 and ECM-receptor interaction signaling pathways. Vascular Inflammation: NF-κB pathway: Activation of NF-κB pathway and upregulation of pro-inflammatory genes. Proteolysis of extracellular matrix components, such as integrin–fibronectin-binding factors, cytokines, immunoglobulin, and complement components. Atherosclerosis: ApoB-100 degradation, LDL aggregation, generation of foam cells, and enhanced lipid deposits. Cardiomyocyte apoptosis by activating Bax protein, a pro-apoptotic protein through cleavage at its Arg34 site; agglutinate and hemolyze erythrocytes. Invading host cells and destroying them through pathological mechanisms of avoiding host immune defenses and antibiotic resistance. Common pathway: Increased intracellular reactive oxygen species (ROS) production, antioxidant depletion, reduction of nitric oxide (NO), activation of the NF-κB pathway, and upregulation of the expression of IL-1β and TNF-α, subsequently causing cell apoptosis and endothelial cell death.	[21,22]
*Treponema denticola*	Major outer sheath protein (MSP) and OppA, Periplasmic flagella, Acylated chymotrypsin-like protease complex (CTLP), Dentilisin Leucine-rich-repeat A: Cystalysin: Lipooligosaccharide: Peptidoglycan Metabolites: SCFAs, Ammonia, Bioactive lipids, Reactive oxygen species (ROS), Proteolytic enzymes	Adherence to fibronectin, plasminogen, and laminin; locomotive movement. Interactions with other oral micro-organisms, synergy in microbial community, and host tissue pathogenesis. Induced immune response via the TLR2/4-MyD88 pathway. Activate the innate immune system through the TLR2 pathway. Upregulation of the TLR2/MyD88-dependent pathways; upregulation of MMPs in hPDL cells. Increase pro-inflammatory cytokines, activation of endothelial cells, and excessive induction of adhesion, cytokines, growth factors, and vasoconstrictors and cell apoptosis. Ligand for coaggregation with *T. forsythia*, involves hemolysis and hemooxidizing hemoglobin Cysteine desulfhydrase activity, activates innate immunity via TLR4, induction of TNF-a and nitric oxide from mice macrophages, induction of IL-1b, IL-6, IL-8, MCP-1, nitric oxide, MMP-8 and PGE2 from human gingival fibroblasts. Induces release of IL-1b, IL-6, IL-8, TNF-a, RANTES, MMP-8, and PGE2 contribute to inflammation and oxidative stress.	[23,24]
*Tannerella forsythia*	S-layer proteins, Surface lipoproteins, *BspA*,	Adhesion and invasion to host cells, serve as ligands for lectin-like receptors present on coaggregating bacteria *F. nucleatum*. Activate pro-inflammatory cytokines and induce cellular apoptosis, activate the NF-κB pathway and apoptotic cell death. Atherosclerosis: Foam cell formation, downregulation of LXRα, LXRβ, and ABCA1, increase in serum CRP and LDL, decrease in serum HDL.	[25]
*Fusobacterium nucleatum*	FadA, GroEL	Binds to VE-cadherin, leading to increased endothelial permeability, disrupted cell function, impaired vascularization, and subsequent bacterial invasion and inflammation of endothelial cells. Atherosclerosis: Decreased serum HDL levels, elevated serum CRP, IL-6, and LDL levels, and foam cell formation. Pro-inflammatory Response: Induction of pro-inflammatory molecules (MCP-1, IL-8) and adhesion molecules (ICAM-1, VCAM-1) in endothelial cells through the TLR-MyD88-NF-κB signaling pathway. Cross-reactivity of Antibodies: Antibodies against bacterial GroEL may cross-react with hHSP60 on endothelial cells, leading to endothelial dysfunction and promoting the development of atherosclerosis.	[23,24,26]

## Data Availability

No new data were created or analyzed in this study. Data sharing is not applicable to this article.

## Supplementary Materials

The following supporting information can be downloaded at: https://www.mdpi.com/article/10.3390/dj13030098/s1, Table S1: Risk of Bias Assessment using GRADE (Grading of Recommendations Assessment, Development, and Evaluation) system.

## Abbreviations

The following abbreviations are used in this manuscript:

PD	Periodontal disease
CVD	Cardiovascular disease
NO	Nitric oxide
ROS	Reactive oxygen species
CDC	Centers for Disease Control and Prevention
LPS	Lipopolysaccharides
GCF	Gingival crevicular fluid
MMP	Matrix metalloproteinases
MIP	Macrophage inflammatory protein
TIMP	Tissue inhibitor of metalloproteinases
Nrf2	Nuclear factor erythroid 2-related factor 2
COPD	Chronic obstructive pulmonary disease
CHD	Coronary heart disease
CRP	C-reactive protein
TLRs	Toll-like receptors
AS	Atherosclerosis
IM-PEDE	Inflammation-Mediated-Polymicrobial-Emergence and Dysbiotic-Exacerbation
ECM	Extracellular matrix
MCP	Monocyte chemoattractant protein
IL	Interleukin
TNF	Tumor necrosis factor
IFN	Interferon
BH4	Tetrahydrobiopterin

## References

[1] Perera M., Al-Hebshi N.N., Speicher D.J., Perera I., Johnson N.W. (2016). Emerging role of bacteria in oral carcinogenesis: A review with special reference to perio-pathogenic bacteria. J. Oral Microbiol..

[2] Hou K., Wu Z.X., Chen X.Y., Wang J.Q., Zhang D., Xiao C., Zhu D., Koya J.B., Wei L., Li J. (2022). Microbiota in health and diseases. Signal Transduct. Target. Ther..

[3] Chow Y.C., Yam H.C., Gunasekaran B., Lai W.Y., Wo W.Y., Agarwal T., Ong Y.Y., Cheong S.L., Tan S.-A. (2022). Implications of *Porphyromonas gingivalis* peptidyl arginine deiminase and gingipain R in human health and diseases. Front. Cell. Infect. Microbiol..

[4] Corredor Z., Suarez-Molina A., Fong C., Cifuentes-C L., Guauque-Olarte S. (2022). Presence of periodontal pathogenic bacteria in blood of patients with coronary artery disease. Sci. Rep..

[5] Schenkein H.A., Papapanou P.N., Genco R., Sanz M. (2020). Mechanisms underlying the association between periodontitis and atherosclerotic disease. Periodontology 2000.

[6] Peng X., Cheng L., You Y., Tang C., Ren B., Li Y., Xu X., Zhou X. (2022). Oral microbiota in human systematic diseases. Int. J. Oral Sci..

[7] Page M.J., McKenzie J.E., Bossuyt P.M., Boutron I., Hoffmann T.C., Mulrow C.D., Shamseer L., Tetzlaff J.M., Akl E.A., Brennan S.E. (2021). The PRISMA 2020 statement: An updated guideline for reporting systematic reviews. BMJ.

[8] O’Dwyer M.C., Furgal A., Furst W., Ramakrishnan M., Capizzano N., Sen A., Klinkman M. (2023). The Prevalence of Periodontitis Among US Adults with Multimorbidity Using NHANES Data 2011-2014. J. Am. Board Fam. Med..

[9] Helmi M.F., Huang H., Goodson J.M., Hasturk H., Tavares M., Natto Z.S. (2019). Prevalence of periodontitis and alveolar bone loss in a patient population at Harvard School of Dental Medicine. BMC Oral Health.

[10] Jiao J., Jing W., Si Y., Feng X., Tai B., Hu D., Lin H., Wang B., Wang C., Zheng S. (2021). The prevalence and severity of periodontal disease in Mainland China: Data from the Fourth National Oral Health Survey (2015-2016). J. Clin. Periodontol..

[11] Abbas Y., Elsaadany B., Ghallab N. (2023). Prevalence of different stages of periodontal diseases among a sample of young adult obese Egyptian patients: A hospital based Cross-sectional study over 1 year. BMC Oral Health.

[12] Varma S.V., Varghese S., Nair S.V. (2023). Prevalence of Chronic Periodontitis and Chronic Stress in the South Indian Population. Cureus.

[13] Morales-Ruiz P., Moreno-Barrera A.M., Ribas-Pérez D., Rodríguez-Menacho D., Flores-Fraile J.M., Gómez-Salgado J., Castaño-Seiquer A. (2023). Periodontal health of a low socioeconomic level population in Yucatan (Mexico): A cross-sectional study. Medicine.

[14] Relvas M., López-Jarana P., Monteiro L., Pacheco J.J., Braga A.C., Salazar F. (2022). Study of Prevalence, Severity and Risk Factors of Periodontal Disease in a Portuguese Population. J. Clin. Med..

[15] Teza H., Pattanateepapon A., Lertpimonchai A., Vathesatogkit P., McKay G.J., Attia J., Thakkinstian A. (2023). Development of Risk Prediction Models for Severe Periodontitis in a Thai Population: Statistical and Machine Learning Approaches. JMIR Form. Res..

[16] Beukers N.G., van der Heijden G.J., van Wijk A.J., Loos B.G. (2017). Periodontitis is an independent risk indicator for atherosclerotic cardiovascular diseases among 60 174 participants in a large dental school in the Netherlands. J. Epidemiol. Community Health.

[17] Lazureanu P.C., Popescu F.G., Stef L., Focsa M., Vaida M.A., Mihaila R. (2022). The Influence of Periodontal Disease on Oral Health Quality of Life in Patients with Cardiovascular Disease: A Cross-Sectional Observational Single-Center Study. Medicina.

[18] Bengtsson V.W., Persson G.R., Berglund J.S., Renvert S. (2021). Periodontitis related to cardiovascular events and mortality: A long-time longitudinal study. Clin. Oral Investig..

[19] Byun S.H., Lee S., Kang S.H., Choi H.G., Hong S.J. (2020). Cross-Sectional Analysis of the Association between Periodontitis and Cardiovascular Disease Using the Korean Genome and Epidemiology Study Data. Int. J. Environ. Res. Public Health.

[20] Tiensripojamarn N., Lertpimonchai A., Tavedhikul K., Udomsak A., Vathesatogkit P., Sritara P., Charatkulangkun O. (2021). Periodontitis is associated with cardiovascular diseases: A 13-year study. J. Clin. Periodontol..

[21] Sato N., Matsumoto T., Kawaguchi S., Seya K., Matsumiya T., Ding J., Aizawa T., Imaizumi T. (2022). *Porphyromonas gingivalis* lipopolysaccharide induces interleukin-6 and c-c motif chemokine ligand 2 expression in cultured hCMEC/D3 human brain microvascular endothelial cells. Gerodontology.

[22] Xie M., Tang Q., Yu S., Sun J., Mei F., Zhao J., Chen L. (2020). *Porphyromonas gingivalis* disrupts vascular endothelial homeostasis in a TLR-NF-kappaB axis dependent manner. Int. J. Oral Sci..

[23] Goetting-Minesky M.P., Godovikova V., Fenno J.C. (2021). Approaches to Understanding Mechanisms of Dentilisin Protease Complex Expression in Treponema denticola. Front. Cell. Infect. Microbiol..

[24] Chukkapalli S.S., Rivera M.F., Velsko I.M., Lee J.Y., Chen H., Zheng D., Bhattacharyya I., Gangula P.R., Lucas A.R., Kesavalu L. (2014). Invasion of oral and aortic tissues by oral spirochete Treponema denticola in ApoE(-/-) mice causally links periodontal disease and atherosclerosis. Infect. Immun..

[25] Chinthamani S., Settem R.P., Honma K., Kay J.G., Sharma A. (2017). Macrophage inducible C-type lectin (Mincle) recognizes glycosylated surface (S)-layer of the periodontal pathogen *Tannerella forsythia*. PLoS ONE.

[26] Lee H.R., Jun H.K., Choi B.K. (2014). *Tannerella forsythia* BspA increases the risk factors for atherosclerosis in ApoE(-/-) mice. Oral Dis..

[27] Duran-Pinedo A.E., Chen T., Teles R., Starr J.R., Wang X., Krishnan K., Frias-Lopez J. (2014). Community-wide transcriptome of the oral microbiome in subjects with and without periodontitis. ISME J..

[28] Gilowski L., Wiench R., Płocica I., Krzemiński T.F. (2014). Amount of interleukin-1beta and interleukin-1 receptor antagonist in periodontitis and healthy patients. Arch. Oral Biol..

[29] Reis C., Da Costa A.V., Guimarães J.T., Tuna D., Braga A.C., Pacheco J.J., Arosa F.A., Salazar F., Cardoso E.M. (2014). Clinical improvement following therapy for periodontitis: Association with a decrease in IL-1 and IL-6. Exp. Ther. Med..

[30] Kumaresan D., Balasundaram A., Naik V.K., Appukuttan D.P. (2016). Gingival crevicular fluid periostin levels in chronic periodontitis patients following nonsurgical periodontal treatment with low-level laser therapy. Eur. J. Dent..

[31] Pavlic V., Peric D., Kalezic I.S., Madi M., Bhat S.G., Brkic Z., Staletovic D. (2021). Identification of Periopathogens in Atheromatous Plaques Obtained from Carotid and Coronary Arteries. BioMed Res. Int..

[32] Barbosa De Accioly Mattos M., Peixoto C.B., Amino J.G.d.C., Cortes L., Tura B., Nunn M., Giambiagi-Demarval M., Sansone C. (2023). Coronary atherosclerosis and periodontitis have similarities in their clinical presentation. Front. Oral Health.

[33] Armingohar Z., Jørgensen J.J., Kristoffersen A.K., Abesha-Belay E., Olsen I. (2014). Bacteria and bacterial DNA in atherosclerotic plaque and aneurysmal wall biopsies from patients with and without periodontitis. J. Oral Microbiol..

[34] Jönsson D., Orho-Melander M., Demmer R.T., Engström G., Melander O., Klinge B., Nilsson P.M. (2020). Periodontal disease is associated with carotid plaque area: The Malmö Offspring Dental Study (MODS). J. Intern. Med..

[35] Sima C., Aboodi G.M., Lakschevitz F.S., Sun C., Goldberg M.B., Glogauer M. (2016). Nuclear Factor Erythroid 2-Related Factor 2 Down-Regulation in Oral Neutrophils Is Associated with Periodontal Oxidative Damage and Severe Chronic Periodontitis. Am. J. Pathol..

[36] Tamaki N., Orihuela-Campos R.C., Inagaki Y., Fukui M., Nagata T., Ito H.O. (2014). Resveratrol improves oxidative stress and prevents the progression of periodontitis via the activation of the Sirt1/AMPK and the Nrf2/antioxidant defense pathways in a rat periodontitis model. Free Radic. Biol. Med..

[37] Brown P.M., Kennedy D.J., Morton R.E., Febbraio M. (2015). CD36/SR-B2-TLR2 Dependent Pathways Enhance *Porphyromonas gingivalis* Mediated Atherosclerosis in the Ldlr KO Mouse Model. PLoS ONE.

[38] Cai Y., Kobayashi R., Hashizume-Takizawa T., Kurita-Ochiai T. (2014). *Porphyromonas gingivalis* infection enhances Th17 responses for development of atherosclerosis. Arch. Oral Biol..

[39] Yang J., Wu J., Zhang R., Yao M., Liu Y., Miao L., Sun W. (2017). *Porphyromonas gingivalis* oral infection promote T helper 17/Treg imbalance in the development of atherosclerosis. J. Dent. Sci..

[40] Parvaneh M., Witting P.K., Ku J., Moradi T., Eroglu E., Freedman B., Sutherland G.T., McCorkindale A., Guennewig B., Choowong P. (2021). Periodontitis induces endothelial dysfunction in mice. Sci. Rep..

[41] Omar M., Alexiou M., Rekhi U.R., Lehmann K., Bhardwaj A., Delyea C., Elahi S., Febbraio M. (2023). DNA methylation changes underlie the long-term association between periodontitis and atherosclerotic cardiovascular disease. Front. Cardiovasc. Med..

[42] Shiheido Y., Maejima Y., Suzuki J.-I., Aoyama N., Kaneko M., Watanabe R., Sakamaki Y., Wakayama K., Ikeda Y., Akazawa H. (2016). *Porphyromonas gingivalis*, a periodontal pathogen, enhances myocardial vulnerability, thereby promoting post-infarct cardiac rupture. J. Mol. Cell. Cardiol..

[43] Chukkapalli S.S., Rivera-Kweh M.F., Velsko I.M., Chen H., Zheng D., Bhattacharyya I., Gangula P.R., Lucas A.R., Kesavalu L. (2015). Chronic oral infection with major periodontal bacteria *Tannerella forsythia* modulates systemic atherosclerosis risk factors and inflammatory markers. Pathog. Dis..

[44] Zhou L.J., Lin W.Z., Meng X.Q., Zhu H., Liu T., Du L.J., Bai X.B., Chen B.Y., Liu Y., Xu Y. (2023). Periodontitis exacerbates atherosclerosis through *Fusobacterium nucleatum*-promoted hepatic glycolysis and lipogenesis. Cardiovasc. Res..

[45] Chukkapalli S.S., Velsko I.M., Rivera-Kweh M.F., Zheng D., Lucas A.R., Kesavalu L. (2015). Polymicrobial Oral Infection with Four Periodontal Bacteria Orchestrates a Distinct Inflammatory Response and Atherosclerosis in ApoE null Mice. PLoS ONE.

[46] Zhou J., Liu L., Wu P., Zhao L., Wu Y. (2022). *Fusobacterium nucleatum* Accelerates Atherosclerosis via Macrophage-Driven Aberrant Proinflammatory Response and Lipid Metabolism. Front. Microbiol..

[47] Suh J.S., Kim S.Y., Lee S.H., Kim R.H., Park N.-H. (2022). Hyperlipidemia is necessary for the initiation and progression of atherosclerosis by severe periodontitis in mice. Mol. Med. Rep..

[48] Velsko I.M., Chukkapalli S.S., Rivera-Kweh M.F., Chen H., Zheng D., Bhattacharyya I., Gangula P.R., Lucas A.R., Kesavalu L. (2015). *Fusobacterium nucleatum* Alters Atherosclerosis Risk Factors and Enhances Inflammatory Markers with an Atheroprotective Immune Response in ApoE(null) Mice. PLoS ONE.

[49] Farrugia C., Stafford G.P., Gains A.F., Cutts A.R., Murdoch C. (2022). *Fusobacterium nucleatum* mediates endothelial damage and increased permeability following single species and polymicrobial infection. J. Periodontol..

[50] Huang C.Y., Shih C.M., Tsao N.W., Lin Y.W., Shih C.C., Chiang K.H., Shyue S.K., Chang Y.J., Hsieh C.K., Lin F.Y. (2016). The GroEL protein of *Porphyromonas gingivalis* regulates atherogenic phenomena in endothelial cells mediated by upregulating toll-like receptor 4 expression. Am. J. Transl. Res..

[51] Chen W., Alshaikh A., Kim S., Kim J., Chun C., Mehrazarin S., Lee J., Lux R., Kim R., Shin K. (2019). *Porphyromonas gingivalis* Impairs Oral Epithelial Barrier through Targeting GRHL2. J. Dent. Res..

[52] Xu W., Pan Y., Xu Q., Wu Y., Pan J., Hou J., Lin L., Tang X., Li C., Liu J. (2018). *Porphyromonas gingivalis* ATCC 33277 promotes intercellular adhesion molecule-1 expression in endothelial cells and monocyte-endothelial cell adhesion through macrophage migration inhibitory factor. BMC Microbiol..

[53] Bugueno I.M., El-Ghazouani F.Z., Batool F., El Itawi H., Anglès-Cano E., Benkirane-Jessel N., Toti F., Huck O. (2020). *Porphyromonas gingivalis* triggers the shedding of inflammatory endothelial microvesicles that act as autocrine effectors of endothelial dysfunction. Sci. Rep..

[54] Wu Y., Xu W., Hou J., Liu Y., Li R., Liu J., Li C., Tang X., Lin L., Pan Y. (2019). *Porphyromonas gingivalis*-Induced MIF Regulates Intercellular Adhesion Molecule-1 Expression in EA.hy926 Cells and Monocyte-Endothelial Cell Adhesion Through the Receptors CD74 and CXCR4. Inflammation.

[55] Viafara-Garcia S.M., Morantes S.J., Chacon-Quintero Y., Castillo D.M., Lafaurie G.I., Buitrago D.M. (2019). Repeated *Porphyromonas gingivalis* W83 exposure leads to release pro-inflammatory cytokynes and angiotensin II in coronary artery endothelial cells. Sci. Rep..

[56] Czesnikiewicz-Guzik M., Nosalski R., Mikolajczyk T.P., Vidler F., Dohnal T., Dembowska E., Graham D., Harrison D.G., Guzik T.J. (2019). Th1-type immune responses to *Porphyromonas gingivalis* antigens exacerbate angiotensin II-dependent hypertension and vascular dysfunction. Br. J. Pharmacol..

[57] Hirasawa M., Kurita-Ochiai T. (2018). *Porphyromonas gingivalis* Induces Apoptosis and Autophagy via ER Stress in Human Umbilical Vein Endothelial Cells. Mediators Inflamm..

[58] Yang W.W., Guo B., Jia W.Y., Jia Y. (2016). *Porphyromonas gingivalis*-derived outer membrane vesicles promote calcification of vascular smooth muscle cells through ERK1/2-RUNX2. FEBS Open Bio.

[59] Park H.-J., Kim Y., Kim M.K., Park H.R., Kim H.J., Bae S.K., Bae M.K. (2020). Infection of *Porphyromonas gingivalis* Increases Phosphate-Induced Calcification of Vascular Smooth Muscle Cells. Cells.

[60] Liu F., Wang Y., Xu J., Liu F., Hu R., Deng H. (2016). Effects of *Porphyromonas gingivalis* lipopolysaccharide on the expression of key genes involved in cholesterol metabolism in macrophages. Arch. Med. Sci..

[61] Shen S., Sun T., Ding X., Gu X., Wang Y., Ma X., Li Z., Gao H., Ge S., Feng Q. (2024). The exoprotein Gbp of *Fusobacterium nucleatum* promotes THP-1 cell lipid deposition by binding to CypA and activating PI3K-AKT/MAPK/NF-κB pathways. J. Adv. Res..

[62] Wang Q., Zhao L., Xu C., Zhou J., Wu Y. (2019). *Fusobacterium nucleatum* stimulates monocyte adhesion to and transmigration through endothelial cells. Arch. Oral Biol..

[63] Charoensaensuk V., Chen Y.C., Lin Y.H., Ou K.L., Yang L.Y., Lu D.Y. (2021). *Porphyromonas gingivalis* Induces Proinflammatory Cytokine Expression Leading to Apoptotic Death through the Oxidative Stress/NF-kappaB Pathway in Brain Endothelial Cells. Cells.

[64] Sampath C., Okoro E.U., Gipson M.J., Chukkapalli S.S., Farmer-Dixon C.M., Gangula P.R. (2021). *Porphyromonas gingivalis* infection alters Nrf2-phase II enzymes and nitric oxide in primary human aortic endothelial cells. J. Periodontol..

[65] Wu C., Guo S., Niu Y., Yang L., Liu B., Jiang N., Su M., Wang L. (2016). Heat-shock protein 60 of *Porphyromonas gingivalis* may induce dysfunction of human umbilical endothelial cells via regulation of endothelial-nitric oxide synthase and vascular endothelial-cadherin. Biomed. Rep..

[66] Scannapieco F.A., Dongari-Bagtzoglou A. (2021). Dysbiosis revisited: Understanding the role of the oral microbiome in the pathogenesis of gingivitis and periodontitis: A critical assessment. J. Periodontol..

[67] Kinane D.F., Stathopoulou P.G., Papapanou P.N. (2017). Periodontal diseases. Nat. Rev. Dis. Primers.

[68] Salvi G.E., Roccuzzo A., Imber J., Stähli A., Klinge B., Lang N.P. (2023). Clinical periodontal diagnosis. Periodontology 2000.

[69] Papapanou P.N., Sanz M., Buduneli N., Dietrich T., Feres M., Fine D.H., Flemmig T.F., Garcia R., Giannobile W.V., Graziani F. (2018). Periodontitis: Consensus report of workgroup 2 of the 2017 World Workshop on the Classification of Periodontal and Peri-Implant Diseases and Conditions. J. Periodontol..

[70] Hajishengallis G. (2015). Periodontitis: From microbial immune subversion to systemic inflammation. Nat. Rev. Immunol..

[71] Bartold P.M., Van Dyke T.E. (2017). Host modulation: Controlling the inflammation to control the infection. Periodontology 2000.

[72] Moutsopoulos N.M., Konkel J.E. (2018). Tissue-Specific Immunity at the Oral Mucosal Barrier. Trends Immunol..

[73] Wilcox M.E., Charbonney E., D’empaire P.P., Duggal A., Pinto R., Javid A., Dos Santos C., Rubenfeld G.D., Sutherland S., Liles W.C. (2014). Oral neutrophils are an independent marker of the systemic inflammatory response after cardiac bypass. J. Inflamm..

[74] Martínez-García M., Hernández-Lemus E. (2021). Periodontal Inflammation and Systemic Diseases: An Overview. Front. Physiol..

[75] Pan W., Wang Q., Chen Q. (2019). The cytokine network involved in the host immune response to periodontitis. Int. J. Oral Sci..

[76] Hajishengallis G., Korostoff J.M. (2017). Revisiting the Page & Schroeder model: The good, the bad and the unknowns in the periodontal host response 40 years later. Periodontology 2000.

[77] Stadler A.F., Angst P.D.M., Arce R.M., Gomes S.C., Oppermann R.V., Susin C. (2016). Gingival crevicular fluid levels of cytokines/chemokines in chronic periodontitis: A meta-analysis. J. Clin. Periodontol..

[78] Suh J.S., Kim S., Boström K.I., Wang C.-Y., Kim R.H., Park N.-H. (2019). Periodontitis-induced systemic inflammation exacerbates atherosclerosis partly via endothelial-mesenchymal transition in mice. Int. J. Oral Sci..

[79] Meyle J., Dommisch H., Groeger S., Giacaman R.A., Costalonga M., Herzberg M. (2017). The innate host response in caries and periodontitis. J. Clin. Periodontol..

[80] Wang G.P. (2015). Defining functional signatures of dysbiosis in periodontitis progression. Genome Med..

[81] Chin Y.T., Cheng G.Y., Shih Y.J., Lin C.Y., Lin S.J., Lai H.Y., Whang-Peng J., Chiu H.C., Lee S.Y., Fu E. (2017). Therapeutic applications of resveratrol and its derivatives on periodontitis. Ann. N. Y. Acad. Sci..

[82] Olsen I., Taubman M.A., Singhrao S.K. (2016). *Porphyromonas gingivalis* suppresses adaptive immunity in periodontitis, atherosclerosis, and Alzheimer’s disease. J. Oral Microbiol..

[83] Dioguardi M., Crincoli V., Laino L., Alovisi M., Sovereto D., Mastrangelo F., Russo L.L., Muzio L.L. (2020). The Role of Periodontitis and Periodontal Bacteria in the Onset and Progression of Alzheimer’s Disease: A Systematic Review. J. Clin. Med..

[84] Sanz M., del Castillo A.M., Jepsen S., Gonzalez-Juanatey J.R., D’aiuto F., Bouchard P., Chapple I., Dietrich T., Gotsman I., Graziani F. (2020). Periodontitis and cardiovascular diseases: Consensus report. J. Clin. Periodontol..

[85] Song W.P., Bo X.W., Dou H.X., Fan Q., Wang H. (2024). Association between periodontal disease and coronary heart disease: A bibliometric analysis. Heliyon.

[86] Nicholas M., Townsend N., Scarborough P., Rayner M. (2015). Corrigendum to: Cardiovascular disease in Europe 2014: Epidemiological update. Eur. Heart J..

[87] Keser G., Aksu K., Direskeneli H. (2018). Discrepancies between vascular and systemic inflammation in large vessel vasculitis: An important problem revisited. Rheumatology.

[88] Campbell L.A., Rosenfeld M.E. (2015). Infection and Atherosclerosis Development. Arch. Med. Res..

[89] Cho H.J., Shin M., Song Y., Park S., Kim H. (2021). Severe Periodontal Disease Increases Acute Myocardial Infarction and Stroke: A 10-Year Retrospective Follow-up Study. J. Dent. Res..

[90] Litvinov E., Litvinov A. (2024). The Relationship Between Periodontitis, Gingivitis, Smoking, Missing Teeth, Endodontic Infections, Aortic Aneurysm, and Coronary Artery Disease: The 10-Year Results of 25 Patients. Cureus.

[91] Huang X., Xie M., Lu X., Mei F., Song W., Liu Y., Chen L. (2023). The Roles of Periodontal Bacteria in Atherosclerosis. Int. J. Mol. Sci..

[92] Mahendra J., Mahendra L., Nagarajan A., Mathew K. (2015). Prevalence of eight putative periodontal pathogens in atherosclerotic plaque of coronary artery disease patients and comparing them with noncardiac subjects: A case-control study. Indian J. Dent. Res..

[93] Pardo A., Signoriello A., Signoretto C., Messina E., Carelli M., Tessari M., De Manna N.D., Rossetti C., Albanese M., Lombardo G. (2021). Detection of Periodontal Pathogens in Oral Samples and Cardiac Specimens in Patients Undergoing Aortic Valve Replacement: A Pilot Study. J. Clin. Med..

[94] Walkenhorst M.S., Reyes L., Perez G., Progulske-Fox A., Brown M.B., Phillips P.L. (2020). A Uniquely Altered Oral Microbiome Composition Was Observed in Pregnant Rats With *Porphyromonas gingivalis* Induced Periodontal Disease. Front. Cell. Infect. Microbiol..

[95] Zhang J., Xie M., Huang X., Chen G., Yin Y., Lu X., Feng G., Yu R., Chen L. (2021). The Effects of *Porphyromonas gingivalis* on Atherosclerosis-Related Cells. Front. Immunol..

[96] Atarbashi-Moghadam F., Havaei S.R., Hosseini N.S., Behdadmehr G., Atarbashi-Moghadam S. (2018). Periopathogens in atherosclerotic plaques of patients with both cardiovascular disease and chronic periodontitis. ARYA Atheroscler..

[97] Rodean I.P., Lazăr L., Halațiu V.-B., Biriș C., Benedek I., Benedek T. (2021). Periodontal Disease Is Associated with Increased Vulnerability of Coronary Atheromatous Plaques in Patients Undergoing Coronary Computed Tomography Angiography-Results from the Atherodent Study. J. Clin. Med..

[98] Joshi C., Bapat R., Anderson W., Dawson D., Cherukara G., Hijazi K. (2021). Serum antibody response against periodontal bacteria and coronary heart disease: Systematic review and meta-analysis. J. Clin. Periodontol..

[99] Xu W., Zhou W., Wang H., Liang S. (2020). Roles of *Porphyromonas gingivalis* and its virulence factors in periodontitis. Adv. Protein Chem. Struct. Biol..

[100] Smalley J.W., Olczak T. (2017). Heme acquisition mechanisms of *Porphyromonas gingivalis*—Strategies used in a polymicrobial community in a heme-limited host environment. Mol. Oral Microbiol..

[101] Zhang Z., Liu D., Liu S., Zhang S., Pan Y. (2020). The Role of *Porphyromonas gingivalis* Outer Membrane Vesicles in Periodontal Disease and Related Systemic Diseases. Front. Cell. Infect. Microbiol..

[102] Okamura H., Hirota K., Yoshida K., Weng Y., He Y., Shiotsu N., Ikegame M., Uchida-Fukuhara Y., Tanai A., Guo J. (2021). Outer membrane vesicles of *Porphyromonas gingivalis*: Novel communication tool and strategy. Jpn. Dent. Sci. Rev..

[103] Basic A., Dahlén G. (2023). Microbial metabolites in the pathogenesis of periodontal diseases: A narrative review. Front. Oral Health.

[104] Razeghian-Jahromi I., Elyaspour Z., Zibaeenezhad M.J., Hassanipour S. (2022). Prevalence of Microorganisms in Atherosclerotic Plaques of Coronary Arteries: A Systematic Review and Meta-Analysis. Evid. Based Complement. Altern. Med..

[105] Akhi R., Wang C., Nissinen A., Kankaanpää J., Bloigu R., Paju S., Mäntylä P., Buhlin K., Sinisalo J., Pussinen P. (2019). Salivary IgA to MAA-LDL and Oral Pathogens Are Linked to Coronary Disease. J. Dent. Res..

[106] Medina-Leyte D.J., Zepeda-García O., Domínguez-Pérez M., González-Garrido A., Villarreal-Molina T., Jacobo-Albavera L. (2021). Endothelial Dysfunction, Inflammation and Coronary Artery Disease: Potential Biomarkers and Promising Therapeutical Approaches. Int. J. Mol. Sci..

[107] Fan Z., Tang P., Li C., Yang Q., Xu Y., Su C., Li L. (2023). *Fusobacterium nucleatum* and its associated systemic diseases: Epidemiologic studies and possible mechanisms. J. Oral Microbiol..

[108] Alonso-Pineiro J.A., Gonzalez-Rovira A., Sánchez-Gomar I., Moreno J.A., Durán-Ruiz M.C. (2021). Nrf2 and Heme Oxygenase-1 Involvement in Atherosclerosis Related Oxidative Stress. Antioxidants.

[109] Channon K.M. (2021). Tetrahydrobiopterin and Nitric Oxide Synthase Recouplers. Handbook of Experimental Pharmacology.

[110] Gangula P., Ravella K., Chukkapalli S., Rivera M., Srinivasan S., Hale A., Channon K., Southerland J., Kesavalu L. (2015). Polybacterial Periodontal Pathogens Alter Vascular and Gut BH4/nNOS/NRF2-Phase II Enzyme Expression. PLoS ONE.

