# Supplementary material for: Unveiling the Molecular Crosstalk Between Periodontal and Cardiovascular Diseases: A Systematic Review
Source: Dent J (Basel). 2025 Feb 25;13(3):98. doi: 10.3390/dj13030098 (PMC11941040; doi:10.3390/dj13030098)
Supplement: Supplementary file 1 [file dentistry-13-00098-s001.zip › dentistry-3445783-supplementary.pdf]

Electronic Search Strategy

Database: sciencedirect.com

Search Date: September 4th 2024

Search Terms and Strategy:

1. Concept of Interest
"Periodontal disease" AND “cardiovascular disease” AND “underlying mechanism”

2. Complete Search Strategy:

- **Years:** 2014 - 2024
- **Article type**: Review articles and research articles
- **Subject areas**:

I. Medicine and Dentistry
II. Biochemistry, Genetics and Molecular Biology
III. Pharmacology, toxicology and pharmaceutical science
IV. Immunology and microbiology
V. Agricultural and biological sciences
VI. Neuroscience
VII. Chemistry
VIII. Environmental Science
IX. Nursing and Health Professions

3. Search Result:

- Studies included: ScienceDirect returned articles based on the intersection of the above search terms and filters.
- Total records retrieved: 226 articles

Database: PubMed

Search Date: September 4th 2024

Search Terms and Strategy:

1. **Concept of Interest**
   "Periodontal disease" AND “cardiovascular disease” AND “underlying mechanisms”
2. **Complete Search Strategy:**
   Year: 2014 – 2024
   Text Availability: Full text
   Article Type:

I. Clinical Study
II. Clinical Trial
III. Comparative Study
IV. Meta-Analysis
V. Multicenter Study
VI. Observational Study
VII. Randomized Controlled Trial
VIII. Research Support, N.I.H., Extramural
IX. Research Support, N.I.H. Intramural
X. Research Support, U.S. Gov't, Non-P.H.S.
XI. Research Support, U.S. Gov't, P.H.S.
XII. Research Support, U.S. Gov't
XIII. Validation Study

3. **Search Result:**

- Studies included: Pubmed returned articles based on the above search terms and filters.
- Total records retrieved: 367 articles

Rationale for the Search Strategy:

1. **Concept of Interest**: "periodontal disease", "cardiovascular disease" and "underlying mechanism" are used to capture studies that focus on the condition of interest.
2. **Study Type**: clinical and translational research including *in vivo* and *in vitro* studies were cited in the review.
3. **Limits**: Limiting to English and the last 10 years ensures the studies are recent and accessible.

Table S1. Risk of Bias Assessment using GRADE (Grading of Recommendations Assessment, Development, and Evaluation) system.

Study Reference	Risk of Bias Domains Assessed	Assessment Summary	Overall Risk of Bias
[8]	Study Design, Population Sampling, Outcome Measurement	The study used a cross-sectional design from NHANES, susceptible to recall bias and unmeasured confounders. The population sampling was representative of U.S. adults aged ≥30 years but with potential selection bias. Diagnostic variability could arise from examiner differences.	Moderate
[9]	Study Design, Population Sampling, Outcome Measurement	A retrospective analysis that relied on secondary data, which may be incomplete or inconsistent. The sample size was appropriate, but it was unclear if the population was fully representative of the target demographic. Diagnosis criteria for periodontitis were well-defined but may lack nuance in retrospective application.	Moderate
[10]	Study Design, Population Sampling, Outcome Measurement	Observational study across age groups (35-74 years) vulnerable to confounding due to age differences. The sampling was limited to regional populations in China, affecting generalizability. Age-stratified prevalence was calculated, but diagnostic consistency across groups may vary.	Moderate to high
[11]	Study Design, Population Sampling, Outcome Measurement	Cross-sectional study design introduces potential confounding factors. The sample size (n=314) was small, and the focus on younger adults in Egypt limits generalizability to broader populations. Prevalence estimates relied on self-reported or non-standardized clinical criteria.	Moderate
[12]	Study Design, Population Sampling, Outcome Measurement	An observational study without clear stratification for key demographic variables, which may affect the validity of findings. While the sample size (n=500) was adequate, it was unclear how representative it was of the broader Indian population aged 30–60 years. Diagnostic criteria were clearly applied.	Moderate
[13]	Study Design, Population Sampling, Outcome Measurement	A cross-sectional study that focused only on severe periodontitis, limiting generalizability to less severe cases. The sample was regional (Mexico) and adequate for the context but may lack broader applicability. Diagnostic consistency was not clearly described.	Moderate to high
[14]	Study Design, Population	Cross-sectional study with a large sample size (n=941) in Portugal, which increases reliability.	Moderate

	Sampling, Outcome Measurement	However, potential recall bias and confounding factors due to the study design may reduce the robustness of the findings. Diagnostic methods were standardized and reliable.	
[15]	Study Design, Population Sampling, Outcome Measurement	Cross-sectional study with a large sample size (n=941) in Portugal, which increases reliability. However, potential recall bias and confounding factors due to the study design may reduce the robustness of the findings. Diagnostic methods were standardized and reliable.	Moderate
[16]	Study Design, Population Sampling, Outcome Measurement	Cross-sectional study using electronic health records from ACTA. The sample included hospitalized patients, which may introduce selection bias. The study found that individuals with periodontitis had twice the odds of CVD. Standardized data extraction methods were applied.	Moderate
[17]	Study Design, Population Sampling, Outcome Measurement	Cross-sectional survey conducted among 221 hospitalized patients in Romania aged 46–76. The sample was small and focused on a specific group with existing cardiovascular conditions, limiting generalizability. Age and health bias were present, but the study provided reliable associations.	Moderate to high
[18]	Study Design, Population Sampling, Outcome Measurement	A prospective longitudinal cohort study of older adults (aged ≥60 years) in Sweden, exploring the relationship between periodontitis and ischemic heart disease. Dental records were retrospectively reviewed, potentially introducing record bias. However, the design and follow-up strengthened the validity of findings.	Low to moderate
[19]	Study Design, Population Sampling, Outcome Measurement	A cross-sectional study in South Korea using a large epidemiological dataset (n=173,209), enhancing reliability. The study identified a significant association between periodontitis and ischemic heart disease. Potential reporting bias and unmeasured confounders were noted.	Moderate
[20]	Study Design, Population Sampling, Outcome Measurement	Observational cohort study in Thailand examining mild, moderate, and severe periodontitis over time. The prospective design minimized recall bias, but there was potential misclassification of disease severity. A strong association between severe periodontitis and CHD was observed.	Moderate
[21]	Study Design, Population Sampling, Outcome Measurement	The study employed an in vitro experimental design. The study design allows for controlled assessment of molecular mechanisms underlying P. gingivalis-induced neuroinflammation. The use of multiple parameter measurements enhances the	Moderate

		strength and reliability of the study's research findings. Findings from an in vitro model may not fully represent the complexity of the in vivo environment.	
[22]	Study Design, Population Sampling, Outcome Measurement	An experimental study using both in vivo and in vitro models. The combination of in vivo and in vitro models provides a comprehensive analysis of endothelial dysfunction and provide strong support for the conclusions. The study uses a relatively small sample size in animal experiments (n = 5 per group), which may reduce statistical power and increase variability.	Moderate
[23]	Study Design, Population Sampling, Outcome Measurement	The study uses laboratory-based experimental models, including bacterial mutagenesis and biochemical assays. No clinical or in vivo validation in human subjects, limiting direct applicability to human periodontal disease. The study does not include randomization or blinding, increasing the risk of selection bias.	Moderate
[24]	Study Design, Population Sampling, Outcome Measurement	The study used an animal model to investigate the causal link between *Treponema denticola* oral infection and atherosclerosis development. The design involved a controlled experimental setup with infected and sham-infected groups. Potential biases include the relatively small sample size (n = 24), possible inter-individual variability in response to infection, and the inherent differences between murine and human physiology.	Moderate
[25]	Study Design, Population Sampling, Outcome Measurement	The study used a combination of in vitro experimental models. The use of appropriate controls enhances the study's reliability. Although used model, they may not fully replicate the complexity of primary human immune responses in vivo.	Moderate
[26]	Study Design, Population Sampling, Outcome Measurement	The study employed an experimental design animal model. The inclusion of both in vitro and in vivo approaches strengthens the study's comprehensiveness. The sample size and details of randomization or blinding procedures were not provided, introducing potential for selection and detection bias. Potential confounding factors such as diet, environmental conditions, and genetic variability within the mouse cohort were not discussed.	Moderate to low
[27]	Study Design, Population	A cross-sectional study with sample size of 7 subjects with periodontitis and 6 healthy controls.	Moderate

	Sampling, Outcome Measurement	Small sample size limitation and potential selection bias. Use advanced meta transcriptomic profiling to highlight transcriptional activity differences	
[28]	Study Design, Population Sampling, Outcome Measurement	A cross-sectional study with clear selection criteria and not controlled for potential confounding factors. Adequate sample size (n =80). Using objective outcome measurement and statistical correlation analysis	Moderate
[29]	Study Design, Population Sampling, Outcome Measurement	Observational, cross-sectional and longitudinal (before and after therapy), lack of good control group for post-treatment comparisons and all patients were from on clinic. Used objective clinical and biochemical measurements and appropriate non-parametric statistical analysis	Moderate
[30]	Study Design, Population Sampling, Outcome Measurement	Interventional, Randomized Controlled Trial (RCT) A single examiner, blinded to treatment allocation, recorded clinical parameters. Statistical methods are clearly stated. Blinded outcome assessor, objective ELISA biomarker	Moderate
[35]	Study Design, Population Sampling, Outcome Measurement	Experimental study investigating the effects of *Fusobacterium nucleatum* on endothelial cells and zebrafish models. The study utilized objective measures such as flow cytometry and permeability assays. However, details on blinding and randomization were not specified, which may introduce bias.	Moderate
[36]	Study Design, Population Sampling, Outcome Measurement	Experimental study examining the role of the GroEL protein from *Porphyromonas gingivalis* in atherogenesis using human coronary artery endothelial cells and mouse models. The study employed objective measures, including gene expression analysis and functional assays. The lack of information on blinding and randomization procedures may introduce bias.	Moderate
[51]	Confounding, timing and standardization of interventions and outcome measurement and reliability	The study minimizes bias by employing standardized methodologies for cell culture, bacterial culture, and mouse model generation, ensuring uniformity in interventions and outcome measurements. However, potential biases may occur from confounding variables such as epithelial permeability assays and bacterial detection, which could affect the reliability of findings despite statistical rigor in data analysis.	Low to moderate
[31]	Study Design, Population Sampling,	This observational study has risk of bias due to its small sample size (n=58), and potential selection bias due to strict inclusion and exclusion criteria, as	Moderate to high

	Outcome Measurement	well as the lack of blinding in periodontal assessments. Additionally, reliance on a single trained periodontist for periodontal examination and potential confounders such as dietary habits and genetic predisposition, which were not controlled, may impact the study's internal validity.	
[32]	Study Design, Population Sampling, Outcome Measurement	This observational study has minimized the risk of bias with strict inclusion/exclusion criteria, and standardized diagnostic protocols, ensuring internal validity. However, potential selection bias may arise from the single-center design, limiting external validity.	Moderate to low
[37]	Study design, confounding, timing and standardization of interventions and outcome measurement and reliability	The study design minimizes bias through standardized interventions and measurements ensure reliability of the outcomes. However, potential confounders such as genetic background differences and environmental factors influencing microbial colonization and immune response could affect results.	Low
[38]	Study design, confounding, timing and standardization of interventions and outcome measurement and reliability	The study has risk of bias due to probable confounding factors such as randomization was done but without stratification for baseline characteristics, and the timing of interventions (3 times per week for 3 weeks) may not fully mimic chronic infection conditions. Standardization of bacterial preparation and outcome measurement (e.g., flow cytometry, ELISA, and PCR) increases reliability, potential inter-experimental variations and the lack of blinded assessments for lesion quantification and cytokine analysis could introduce bias.	Moderate
[39]	Study Design, Population Sampling, Outcome Measurement	ApoE knockout mouse model for atherosclerosis research. Used various methods to assess immune response.	Moderate
[40]	Study Design, Population Sampling, Outcome Measurement	The study used a controlled experimental design in hyperlipidemic ApoE$^{-/-}$ mice. The use of a control group reduced selection bias, but the absence of blinding in outcome assessments may introduce detection bias. Objective ELISA biomarkers were used for systemic inflammation assessment, ensuring reliable outcome measurement. Some limitations exist in the external validity to human populations.	Moderate

[33]	Study Design, Population Sampling, Outcome Measurement	Case-control study comparing VD patients with and without CP. Objective molecular techniques were used for bacterial identification. Lack of information on blinding introduces potential bias.	Moderate
[41]	Study Design, Population Sampling, Outcome Measurement	Controlled experimental study using mice model. Objective measures such as whole-genome bisulfite sequencing was used. Lack of information on blinding and attrition rates introduces potential bias.	Moderate to low
[34]	Study Design, Population Sampling, Outcome Measurement	Population-based observational study with standardized cardiovascular and periodontal assessments. Lack of information on blinding and voluntary participation may introduce bias.	Moderate
[52]	Confounding, timing and standardization of interventions and outcome measurement and reliability	This study has risk of bias, mainly due to potential confounding factors, e.g. differences in bacterial strain and variations in cell line responses that were not fully reported. Although, interventions were standardized, outcome measurements, factors like bacterial culture conditions and reagent batch variations could still affect the reliability of the results.	Moderate
[53]	Confounding, timing and standardization of interventions and outcome measurement and reliability	Potential bias of this study are confounding factors, such as the lack of randomization in the cell cultures and treatment and the variation in the timing of interventions. There is a lack of standardization in cell culture conditions and assays. There is a risk of bias in the outcomes because certain steps were not independently checked or blinded.	Moderate to high
[42]	Study Design, Population Sampling, Outcome Measurement	Experimental study using C57BL/6J mice inoculated with *Porphyromonas gingivalis* to assess myocardial vulnerability post-myocardial infarction. Standardized procedures were employed, but the study lacks details on blinding and randomization, which may introduce bias.	Moderate
[54]	Confounding, timing and standardization of interventions and outcome measurement and reliability	There could be some bias in this study due to variations in time of interventions and outcomes which might affect the consistency of the results.	Low to moderate
[55]	Confounding, timing and standardization	This study has a potential bias due to confounding factors such as various inflammatory and metabolic pathways affect endothelial responses to *P.*	Moderate to low

	of interventions and outcome measurement and reliability	*gingivalis*. Since the study has well timing and interventions, differences in cell sources and variations between *P. gingivalis* strains could affect the reliability of the outcomes.	
[56]	Confounding, timing and standardization of interventions and outcome measurement and reliability	In this study the risk of bias including confounding factors such as common risk variables for both periodontal disease and hypertension. Additionally, the timing and standardization of interventions and outcome measurements have some limitations, particularly since the study used a peritoneal administration model rather than an oral infection model, which may not fully replicate the natural pathogenesis of periodontal disease.	Moderate
[57]	Confounding, timing and standardization of interventions and outcome measurement and reliability	The risk of bias in this study are confounding factors such as multiple inflammatory and metabolic pathways contribute to endothelial apoptosis, which were not controlled. There is variations in bacterial strain effects and inconsistencies with other studies raise questions on reliability of the outcome.	Moderate to low
[58]	Confounding, timing and standardization of interventions and outcome measurement and reliability	This study describes the evidence linking *P. gingivalis*-derived outer membrane vesicles (OMV) to vascular smooth muscle cell (VSMC). Potential confounding factors include inflammatory mediators or host immune responses, which were not fully addressed. Timing and standardization of interventions were well-defined, however the results in vitro and ex vivo models raise questions about the outcome measurement reliability.	Moderate
[59]	Confounding, timing and standardization of interventions and outcome measurement and reliability	In this experimental study, there is potential risk of confounding factors such as multiple inflammatory and metabolic factors contribute to vascular calcification. Variability in cellular responses and differences from in vivo conditions may impact the reliability and generalizability of the findings.	Moderate
[43]	Study Design, Population Sampling, Outcome Measurement	Experimental study with $ApoE^{-/-}$ mice orally infected with *Tannerella forsythia* to evaluate periodontal disease and systemic effects. The study utilized objective measures but did not specify blinding or randomization methods, potentially introducing bias.	Moderate
[44]	Study Design, Population Sampling,	Experimental study investigating the impact of *Fusobacterium nucleatum* on atherosclerosis in mice. The study employed objective outcome	Moderate

	Outcome Measurement	measures but lacks information on blinding and randomization, which may affect internal validity.	
[45]	Study Design, Population Sampling, Outcome Measurement	Experimental study involving $ApoE^{-/-}$ mice orally infected with a combination of four periodontal bacteria to assess inflammatory responses and atherosclerosis. The study used objective measures but did not detail blinding or randomization procedures, introducing potential bias.	Moderate
[46]	Study Design, Population Sampling, Outcome Measurement	Experimental study examining the effects of *Fusobacterium nucleatum* on atherosclerosis via macrophage-driven inflammatory responses in mice. Objective measures were utilized, but the study lacks details on blinding and randomization, which may introduce bias.	Moderate
[47]	Study Design, Population Sampling, Outcome Measurement	Experimental study assessing the necessity of hyperlipidemia for atherosclerosis progression in mice with severe periodontitis. The study employed objective outcome measures but did not specify blinding or randomization methods, potentially introducing bias.	Moderate
[48]	Study Design, Population Sampling, Outcome Measurement	Experimental study investigating the impact of *Fusobacterium nucleatum* on atherosclerosis risk factors and inflammatory markers in $ApoE^{-/-}$ mice. Objective measures were used, but the study lacks information on blinding and randomization, which may affect internal validity.	Moderate
[60]	Confounding, timing and standardization of interventions and outcome measurement and reliability	The risk of bias in this study could be the potential confounding factors, such as various pathways may influence foam cell formation, all were controlled. The timing and standardization of interventions and outcome measurements were well-defined, but variability in cell sources and inconsistencies with other studies suggest potential reliability of the results.	Moderate to high
[61]	Confounding, timing and standardization of interventions and outcome measurement and reliability	The risk of bias in this study may arise due to potential confounding factors, such as other microbial contributors to atherosclerosis, host immune variations, and environmental influences. Additionally, limitations in timing, standardization of interventions, and outcome measurements may impact the reliability and reproducibility of the findings.	Moderate
[49]	Study Design, Population Sampling,	Experimental study investigating the effects of *Fusobacterium nucleatum* on endothelial cells and zebrafish models. The study utilized objective measures such as flow cytometry and permeability	Moderate

	Outcome Measurement	assays. However, details on blinding and randomization were not specified, which may introduce bias.	
[62]	Confounding, timing and standardization of interventions and outcome measurement and reliability	This study has some potential bias risks, mainly due to confounding factors, as the influence of *F. nucleatum* alongside other periodontal bacteria, which could affect the observed data. Additionally, variations in timing and standardization of interventions over time, may limit the reliability of the results.	Moderate to high
[50]	Study Design, Population Sampling, Outcome Measurement	Experimental study in both in-vitro and in-vivo, examining the role of the GroEL protein from *Porphyromonas gingivalis* in atherogenesis using human coronary artery endothelial cells and mouse models. The study employed objective measures, including gene expression analysis and functional assays. The lack of information on blinding and randomization procedures may introduce bias.	Moderate
[63]	Confounding, timing and standardization of interventions and outcome measurement and reliability	This experimental study carries a moderate risk of bias due to possible confounding factors. Additionally, differences in timing, how interventions were applied, and the consistency of outcome measurements using different cell models may affect the reliability of the findings.	Moderate
[64]	Confounding, timing and standardization of interventions and outcome measurement and reliability	The study provides valuable insights into *Porphyromonas gingivalis*-induced endothelial dysfunction, some bias risks remain. Factors like immune variability, timing of interventions, and lack of standardized infection protocols may affect reproducibility and outcome reliability.	Moderate
[65]	Confounding, timing and standardization of interventions, outcome reliability	Experimental study investigating the effects of *P. gingivalis* HSP60 to HUVECs affecting the protein expression levels of endothelial nitric oxide synthase (eNOS) and vascular endothelial (VE)-cadherin. The potential biases include the in vitro nature of the experiments, which may not fully represent in vivo conditions, and the limited exploration of alternative mechanisms.	Moderate to high
